# Neutrophil extracellular traps regulate ischemic stroke brain injury

**DOI:** 10.1172/JCI154225

**Published:** 2022-05-16

**Authors:** Frederik Denorme, Irina Portier, John L. Rustad, Mark J. Cody, Claudia V. de Araujo, Chieko Hoki, Matthew D. Alexander, Ramesh Grandhi, Mitchell R. Dyer, Matthew D. Neal, Jennifer J. Majersik, Christian C. Yost, Robert A. Campbell

**Affiliations:** 1 Molecular Medicine Program,; 2 Department of Neurology,; 3 Deparment of Radiology and Imaging Sciences, and; 4 Department of Neurosurgery, University of Utah, Salt Lake City, Utah, USA.; 5 Department of Surgery, University of Pittsburgh, Pittsburgh, Pennsylvania, USA.; 6 Department of Pediatrics and; 7 Department of Internal Medicine, University of Utah, Salt Lake City, Utah, USA.

**Keywords:** Hematology, Neutrophils, Platelets, Stroke

## Abstract

Ischemic stroke prompts a strong inflammatory response, which is associated with exacerbated outcomes. In this study, we investigated mechanistic regulators of neutrophil extracellular trap (NET) formation in stroke and whether they contribute to stroke outcomes. NET-forming neutrophils were found throughout brain tissue of ischemic stroke patients, and elevated plasma NET biomarkers correlated with worse stroke outcomes. Additionally, we observed increased plasma and platelet surface–expressed high-mobility group box 1 (HMGB1) in stroke patients. Mechanistically, platelets were identified as the critical source of HMGB1 that caused NETs in the acute phase of stroke. Depletion of platelets or platelet-specific knockout of HMGB1 significantly reduced plasma HMGB1 and NET levels after stroke, and greatly improved stroke outcomes. We subsequently investigated the therapeutic potential of neonatal NET-inhibitory factor (nNIF) in stroke. Mice treated with nNIF had smaller brain infarcts, improved long-term neurological and motor function, and enhanced survival after stroke. nNIF specifically blocked NET formation without affecting neutrophil recruitment after stroke. Importantly, nNIF also improved stroke outcomes in diabetic and aged mice and was still effective when given 1 hour after stroke onset. These results support a pathological role for NETs in ischemic stroke and warrant further investigation of nNIF for stroke therapy.

## Graphical abstract

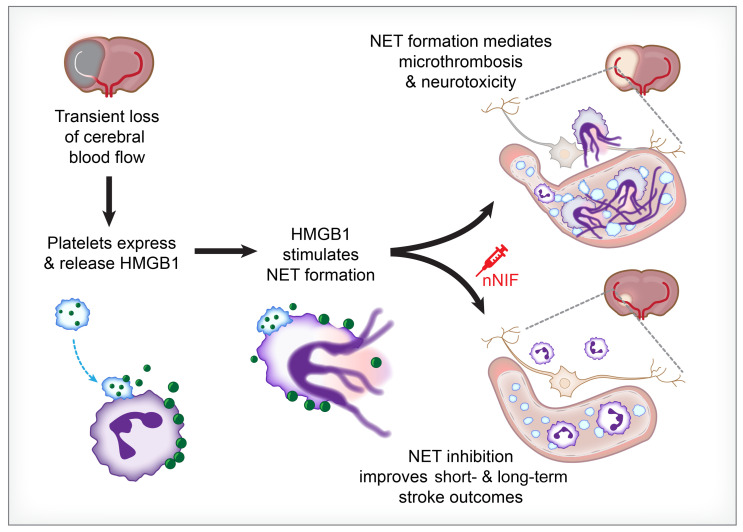

## Introduction

Ischemic stroke is caused by intravascular thrombus formation obstructing cerebral blood flow and affects millions of people every year. Despite improved prophylactic and therapeutic interventions, ischemic stroke is still a major cause of death and disability worldwide (1). Currently, ischemic stroke therapy is aimed at quick restoration of cerebral blood flow. However, few patients are eligible for therapy, and removal of the pathological thrombus does not guarantee improved outcomes, even when treatment is started early (2, 3). In recent years, a multitude of studies have documented that once cerebral blood flow stops, a detrimental cascade of inflammation, microvascular thrombosis, and neurotoxicity takes place in the brain and contributes to ischemic stroke outcomes irrespective of reperfusion therapy (4).

Historically, aberrant thrombus formation was considered to be caused by abnormal blood coagulation and platelet activation, resulting in blood vessel occlusion. Nevertheless, thrombosis also involves other components of the systemic blood circulation, including monocytes and neutrophils (5). The interaction between coagulation, platelets, and the innate immune system has been referred to as immunothrombosis (6). Immunothrombosis is considered an independent process of innate immunity that is specifically aimed at reducing the spread of pathogens in the circulation and culminates in the formation of neutrophil extracellular traps (NETs). NETs are extracellular DNA lattices generated upon excessive neutrophil activation, designed to trap pathogens and help fight infections (7). Notably, NET formation in the bloodstream can trap other blood cells, resulting in pathological thrombus formation including venous thrombosis (8, 9). Several studies have found NETs in ischemic stroke thrombi (10, 11) and surrogate markers of NETs in plasma from ischemic stroke patients (12–14). Similarly, NETs have been reported in brain tissue and plasma of mice subjected to ischemic stroke (15, 16). However, it remains unclear whether NETs are present in brain tissue from stroke patients. Likewise, mechanistic regulators of NET formation in ischemic stroke are currently unknown, and whether NETs play a detrimental role in the acute phase of ischemic stroke has yet to be determined.

Through a translational approach studying ischemic stroke patients and employing murine models of ischemic stroke, we report a key role for platelet-derived high-mobility group box 1 (HMGB1) in the formation of NETs in the acute phase of stroke, resulting in exacerbated stroke outcomes. Treatment with a recently discovered neonatal NET-inhibitory factor (nNIF) reduced ischemic stroke brain injury and stroke-related mortality in mice.

## Results

### NETs are present in brain tissue from ischemic stroke patients.

**Figure 1:**
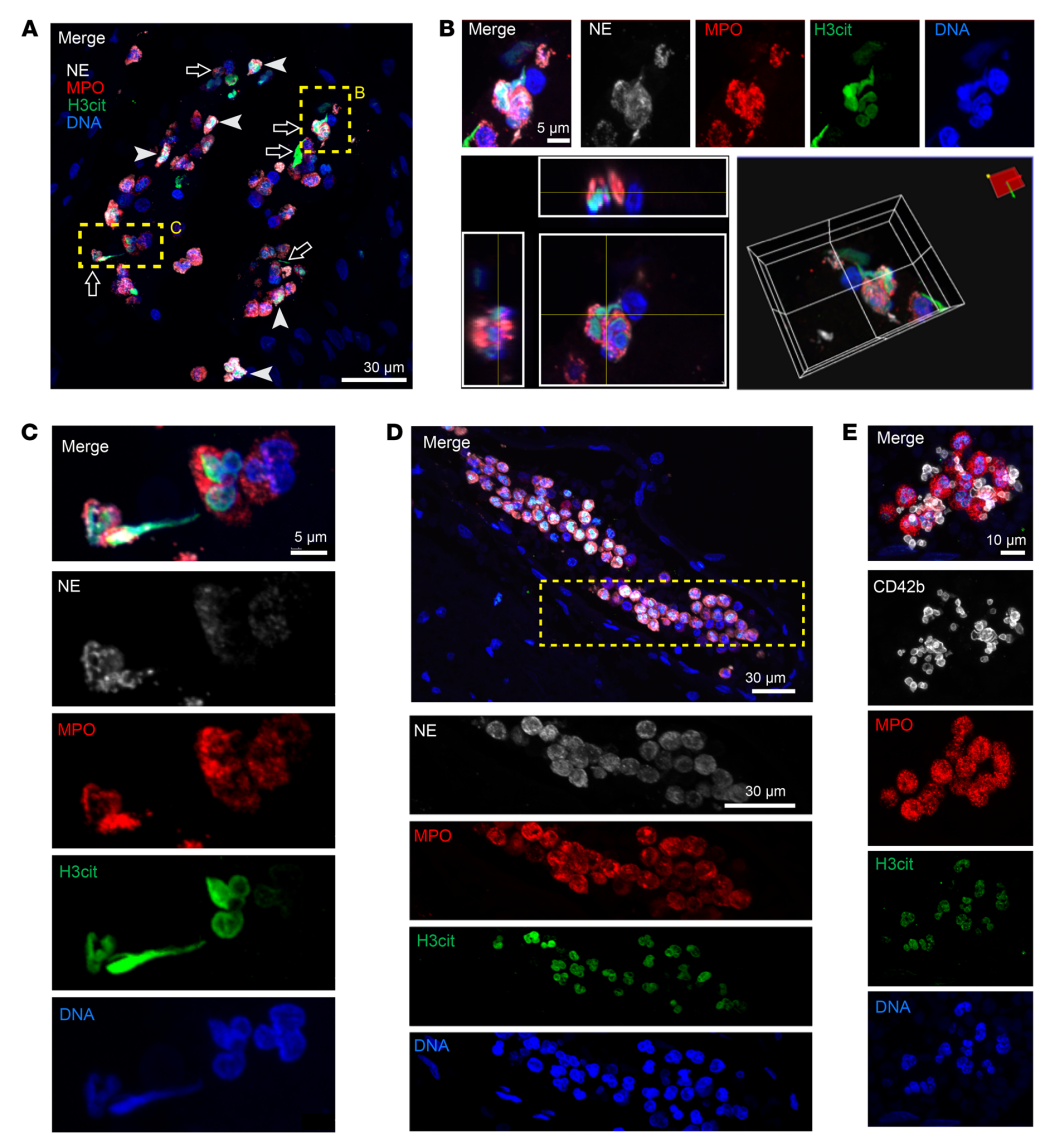
NETs are found in ipsilesional brain tissue from ischemic stroke patients. Ipsilesional brain tissue was obtained from the NIH NeuroBioBank from 3 patients who died after ischemic stroke. (**A**–**D**) NETs were identified by colocalization of myeloperoxidase (MPO; red), neutrophil elastase (NE; white), citrullinated histone H3 (H3cit; green), and DNA (DAPI; blue). (**A**) Neutrophils were in different stages of NET formation, with nuclear decondensation and intracellular H3cit staining (arrowheads) and released extracellular NETs (arrows) frequently observed. Scale bar: 30 μm. (**B**) Top row: High-power magnification image of an extracellular NET with the separate color channels. Bottom left: *z* projection illustrates colocalization of different NET markers. Bottom right: 3D rendering shows H3cit^+^ DNA release from a neutrophil. Scale bar: 5 μm. (**C**) High-power magnification image of 3 neutrophils in different stages of NET formation. Scale bar: 5 μm. (**D**) Example of an intravascular neutrophil-rich thrombus from a separate patient. Scale bars: 30 μm. (**E**) NETs were identified by colocalization of MPO (red), H3cit (green), and DNA (DAPI; blue), and platelets were stained with CD42b (white). Scale bar: 10 μm. Platelets associated with NETs within a vessel in the ipsilesional stroke brain are shown. Images are representative of 2 separate patients (**A**–**C**, patient 1; **D** and **E**, patient 2), representing findings from a total of 3 patients.

To examine whether NETs are present in human brain tissue after ischemic stroke, we obtained brain specimens from 3 patients who died after ischemic stroke. Brain specimens were stained with the neutrophil-specific markers neutrophil elastase (NE) and myeloperoxidase (MPO), as well as with citrullinated histone H3 (H3cit), a marker for neutrophils undergoing NET formation. In agreement with a previous report (17), neutrophils were readily found in ipsilesional brain tissue from all stroke patients (Figure 1 and Supplemental Figure 1; supplemental material available online with this article; https://doi.org/10.1172/JCI154225DS1), while neutrophils were mostly absent in contralateral brain tissue from stroke patients (Supplemental Figure 2). Several neutrophils (NE and MPO double-positive cells) in the ipsilesional brain tissue stained positive for H3cit, indicating they either were in the process of NET formation or had already formed a NET (Figure 1 and Supplemental Figure 1). In all patients, both extracellular NETs (arrows) and neutrophils undergoing NET formation (arrowheads) were found (Figure 1, A–D, Supplemental Figure 1, and Supplemental Video 1). In all cases, NE, MPO, DNA, and citrullinated histones colocalized with each other (Figure 1 and Supplemental Figure 1). Neutrophils in the contralesional hemisphere did not stain for H3cit (Supplemental Figure 2).

In several patients, H3cit^+^ neutrophils were found in cerebral blood vessels, trapped in neutrophil-rich microthrombi occasionally occluding the vessel lumen (Figure 1, D and E, and Supplemental Figure 1D). To investigate the contribution of platelets to this phenomenon, we stained consecutive brain slides for DNA, MPO, H3cit, and CD42b, a platelet-specific marker. As expected, platelets bridged neutrophils in these microthrombi, implying a role for platelet-neutrophil interactions in mediating microthrombus formation in clinical ischemic stroke (Figure 1E). Interestingly, many of the neutrophils found in contact with platelets were undergoing NET formation, suggesting that platelets participate in NET release. Consistent with these findings, we observed NETs predominantly in platelet-rich areas of stroke thrombi retrieved from patients undergoing thrombectomy (Supplemental Figure 3). Notably, as in brain tissue, all MPO^+^ cells also stained for NE in stroke patient thrombi (Supplemental Figure 4), implying that MPO in thrombi and brain tissue is mainly of neutrophil origin.

### Plasma markers of immunothrombosis are increased in ischemic stroke patients, and NET markers correlate with stroke outcomes.

**Figure 2:**
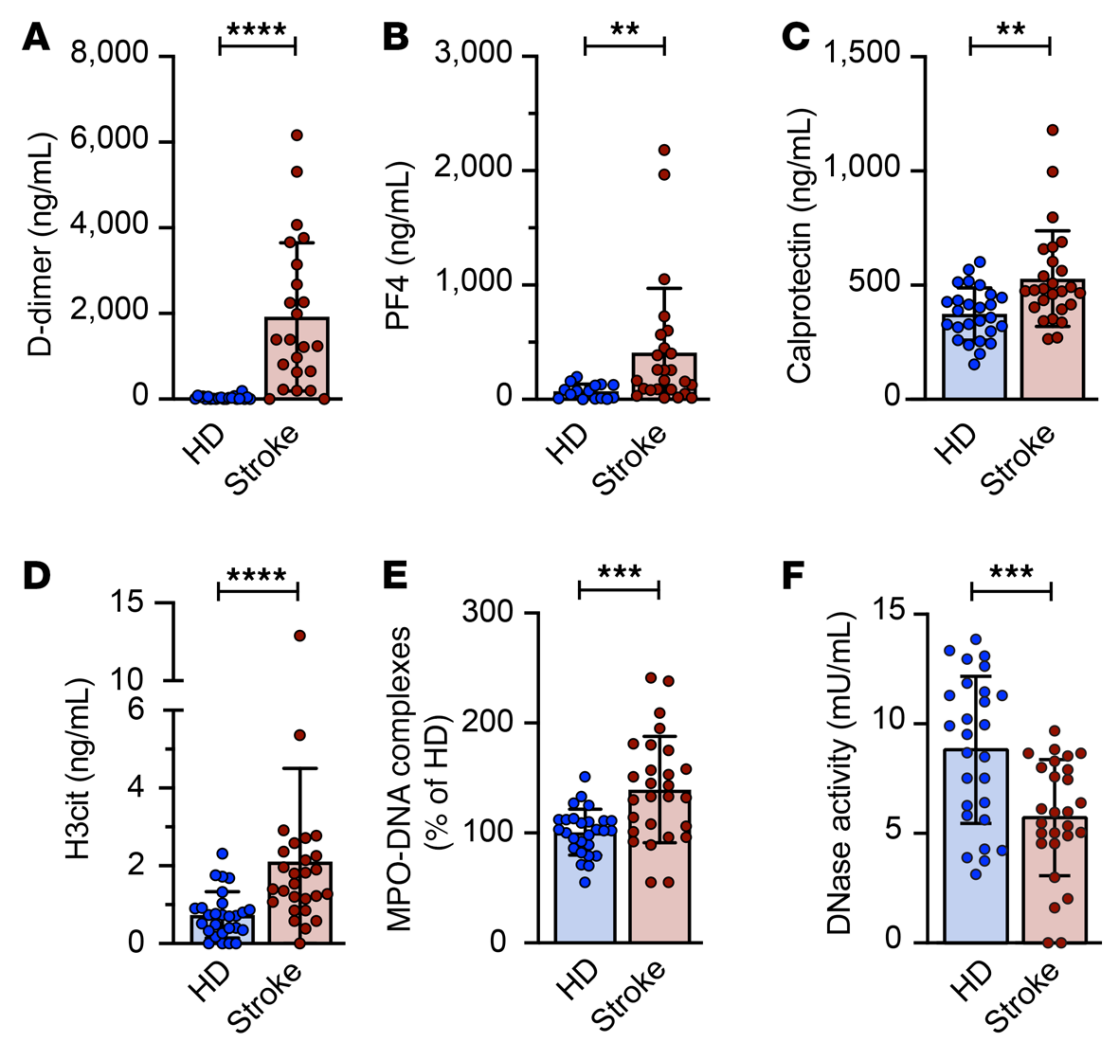
Plasma markers of immunothrombosis are increased in ischemic stroke patients. Plasma samples were obtained within 48 hours of hospital admission from stroke patients or age- and sex-matched healthy donors (HD). D-dimer (**A**), PF4 (**B**), neutrophil calprotectin (**C**), H3cit (**D**), MPO-DNA complexes (**E**), and DNase activity (**F**) were measured by ELISA. *n =* 27 per group. Groups were compared by Mann-Whitney test. **P < 0.01, ***P < 0.001, ****P < 0.0001.

To further address immunothrombosis in ischemic stroke, we measured markers of coagulation (D-dimer; Figure 2A), platelet activation (platelet factor 4 [PF4]; Figure 2B), neutrophil activation (calprotectin; Figure 2C), NET formation (H3cit, Figure 2D; and MPO-DNA complexes, Figure 2E), and NET degradation potential (DNase activity; Figure 2F) in plasma from matched healthy donors and stroke patients (Supplemental Table 1). Markers of immunothrombosis were significantly elevated and plasma DNase activity was greatly reduced in ischemic stroke patients compared with healthy donors (Figure 2). We next used a Spearman correlation analysis to investigate whether markers of immunothrombosis correlated with either stroke severity at admission or stroke outcomes at discharge (Supplemental Table 2). Stroke severity at admission did not correlate with immunothrombosis biomarkers. Interestingly, only markers of NET formation correlated with ischemic stroke outcomes (modified Rankin scale at discharge). Both plasma H3cit and MPO-DNA levels positively correlated with stroke outcomes (*r* = 0.45, *P =* 0.024, and *r* = 0.507, *P =* 0.01, respectively). Sex, comorbidities, thrombolysis, use of antiplatelet drugs or anticoagulation drugs, or ischemic stroke etiology had no effect on plasma NET levels in our cohort (Supplemental Figure 5).

### Platelet-neutrophil interactions mediate NET formation through HMGB1.

**Figure 3:**
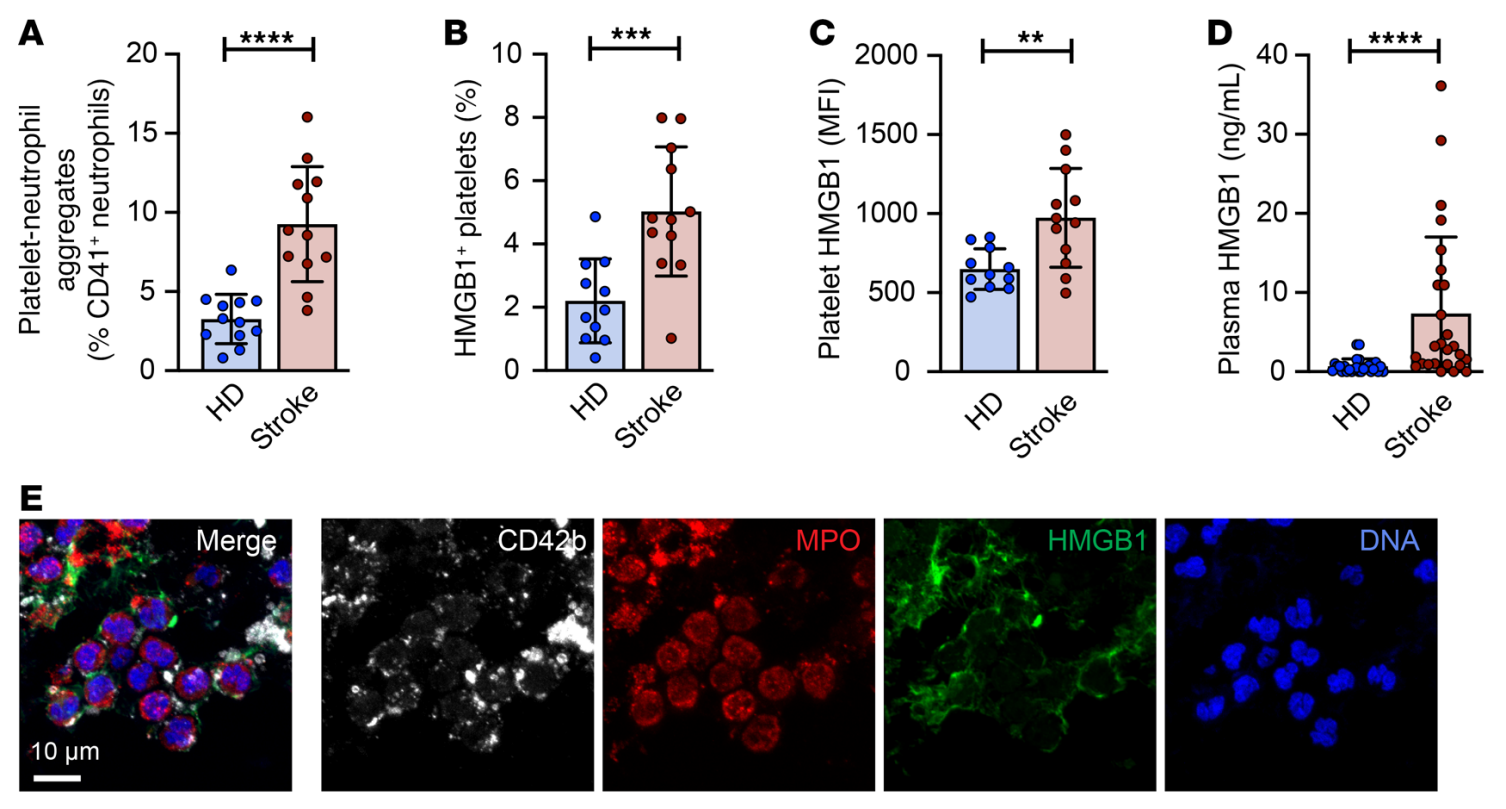
Platelet HMGB1 is increased in ischemic stroke patients and is found at the interface between platelets and neutrophils in ischemic stroke thrombi. (**A**–**C**) Flow cytometry was performed on diluted whole blood (**A**) and washed platelets (**B** and **C**) isolated from ischemic stroke patients and matched healthy donors (HD) to quantify platelet-neutrophil aggregates and platelet HMGB1 expression levels, respectively. *n =* 11–12 per group. (**D**) Plasma HMGB1 levels were measured by ELISA in ischemic stroke patients and matched healthy donors. *n =* 27 per group. (**E**) Ischemic stroke patient thrombi were stained for platelets (CD42b, white), HMGB1 (green), neutrophils (MPO, red) and DNA (DAPI, blue). Scale bar: 10 μm. Image is representative of 7 ischemic stroke thrombi. Groups were compared by unpaired *t* test (**A**–**C**) or Mann-Whitney test (**D**). **P < 0.01, ***P < 0.001, ****P < 0.0001.

Platelets are known for their role in NET formation (6), and we observed NETs predominantly in platelet-rich areas of ischemic stroke thrombi as well as in microthrombi in the brain (Figure 1E and Supplemental Figure 3). Additionally, plasma levels of PF4 correlated with both plasma H3cit and MPO-DNA levels in our cohort (*r* = 0.376, *P =* 0.024, and *r* = 0.556, *P =* 0.001, respectively), implying a role for platelet activation in stroke-induced NET formation. To examine whether platelets could contribute to neutrophil activation and NET formation, we first examined platelet-neutrophil aggregate levels in ischemic stroke patients. Consistent with previous studies (18), circulating platelet-neutrophil aggregates were significantly increased in stroke patients (Figure 3A and Supplemental Figure 6). As platelets interact with neutrophils to induce NET formation through different mechanisms, such as danger-associated molecular patterns (DAMPs), including HMGB1 (19, 20), we examined whether platelet and plasma HMGB1 levels increased during ischemic stroke. Platelets from stroke patients had significantly elevated HMGB1 surface expression (Figure 3, B and C) and increased plasma HMGB1 levels (Figure 3D) compared with healthy donors. Furthermore, HMGB1 predominantly localized at the interface of platelets interacting with neutrophils in ischemic stroke thrombi (Figure 3E). We next examined whether plasma HMGB1 correlated with plasma NET markers and observed that HMGB1 significantly correlated with H3cit and MPO-DNA (*r* = 0.433, *P =* 0.0019, and *r* = 0.3889, *P =* 0.0057, respectively).

### Platelets drive HMGB1 release and NET formation, exacerbating stroke outcomes.

**Figure 4:**
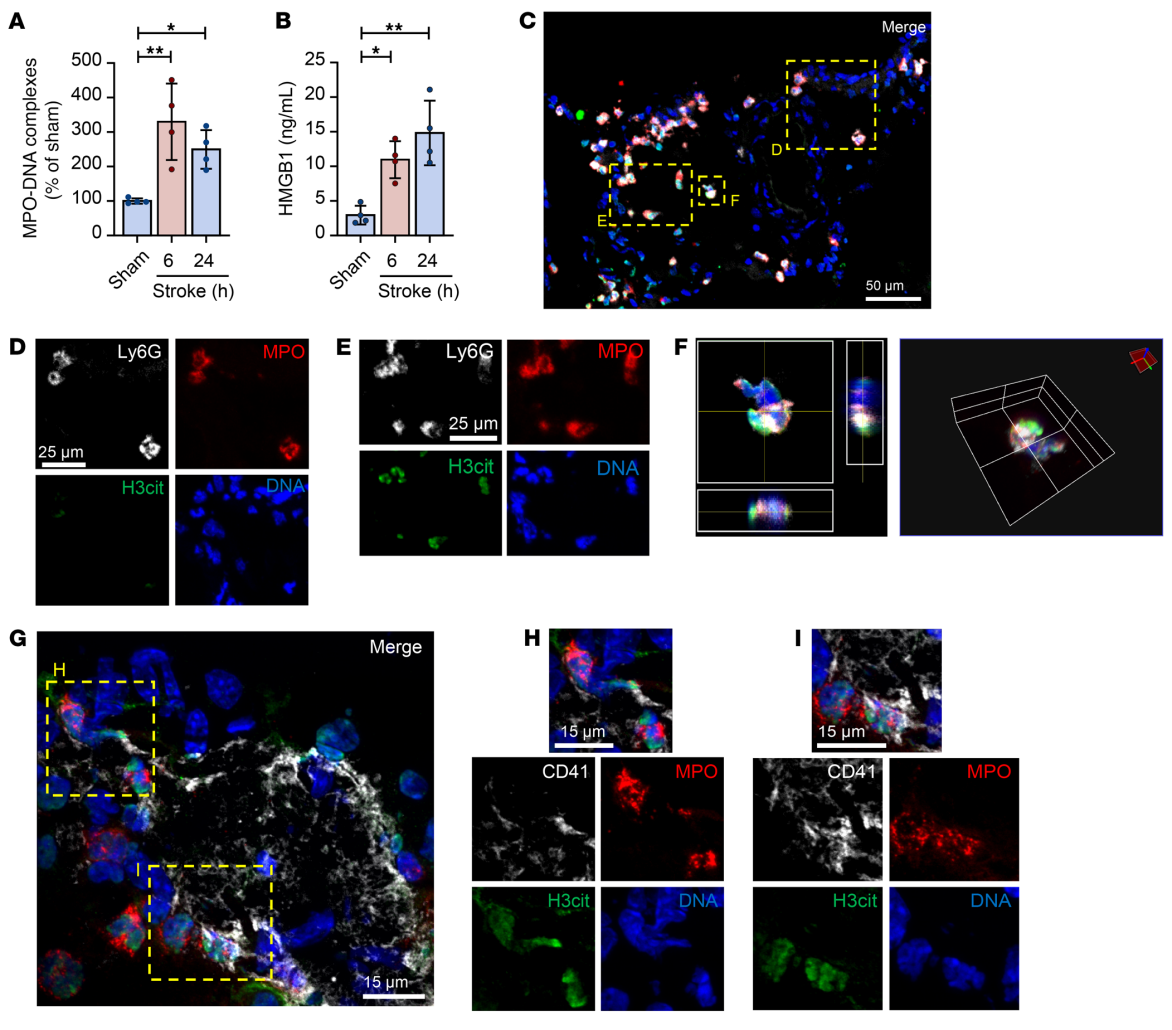
Characterization of NET formation in a mouse model of ischemic stroke. Mice were subjected to 1 hour of tMCAO or sham surgery. Plasma was isolated 6 or 24 hours after stroke onset, and brains were collected and processed for histology. (**A**) MPO-DNA complexes were measured using an in-house MPO-DNA ELISA. *n =* 4 per group. (**B**) Plasma HMGB1 levels were measured by ELISA. *n =* 4 per group. (**C**) Brain tissue was stained for the presence of NETs by a combination of Ly6G (white), MPO (red), H3cit (green), and DNA (DAPI, blue). Scale bar: 50 μm. (**D**–**F**) Both neutrophils not undergoing NET formation (H3cit^–^, highlighted in **D**) and neutrophils undergoing NET formation (H3cit^+^, highlighted in **E** and **F**) were observed. Scale bars: 25 μm. (**G**–**I**) Brain tissue was stained for the colocalization of NETs with platelets by a combination of MPO (red), H3cit (green), platelets (CD41, white), and DNA (DAPI, blue). Scale bars: 15 μm. Images are representative of 4 mice. Groups were compared by ordinary 1-way ANOVA. *P < 0.05, **P < 0.01.

To examine the role of platelet HMGB1 in vivo, we used a murine transient middle cerebral artery occlusion (tMCAO) model, in which the right MCA is occluded for 1 hour followed by 23 hours of reperfusion. Using this model, we observed a significant increase in circulating levels of HMGB1 and MPO-DNA complexes 6 and 24 hours after stroke in comparison with sham animals (Figure 4, A and B), consistent with our observations in human ischemic stroke patients. In addition, we readily detected NETs in the brain throughout the ischemic hemisphere 6 and 24 hours after stroke onset (Figure 4, C and D, and Supplemental Figure 7), while NETs and neutrophils were not observed in the contralateral hemisphere (data not shown). As in stroke patients, we observed 3 types of neutrophils (Ly6G and MPO double positive): (a) H3cit-negative, non-NETting neutrophils; (b) extracellular NETs; and (c) neutrophils in the early stages of NET formation (Figure 4C). Furthermore, platelet-neutrophil complexes were frequently observed in stroke brain tissue, and platelets colocalized with NETs (Figure 4D). Importantly, staining in brains from mice after tMCAO was similar to that observed in human ischemic stroke patients (compare Figure 1 with Figure 4).

**Figure 5:**
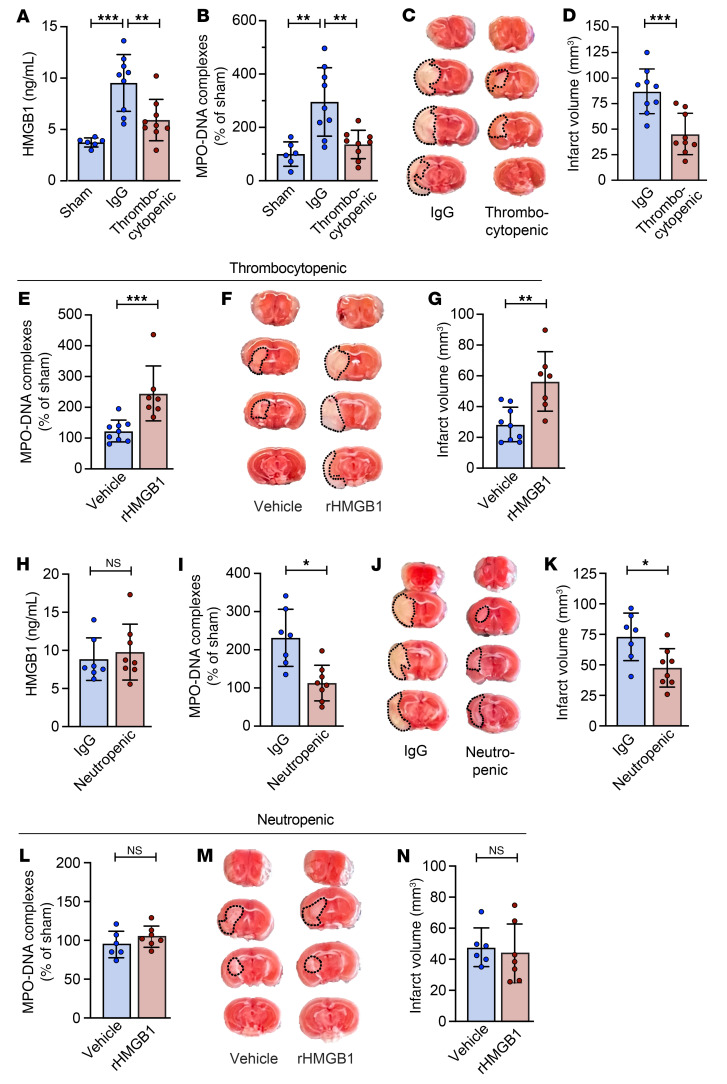
Platelets mediate HMGB1 release, contributing to detrimental NET formation in a mouse model of ischemic stroke. Mice were subjected to 1 hour of tMCAO followed by 23 hours of reperfusion or sham surgery. Plasma was isolated and brains were analyzed for ischemic stroke brain damage by TTC staining 24 hours after stroke onset. Upon TTC staining, live brain tissue will stain red, while dead brain tissue will remain white (outlined with black dotted line). (**A**–**D**) Immediately after stroke onset, mice were injected with a platelet-depleting antibody or IgG control. Twenty-four hours later, plasma HMGB1 (**A**) and MPO-DNA complexes (**B**) were assessed as well as brain infarct volume (**C** and **D**). *n =* 6 for sham mice; *n =* 9 for groups subjected to stroke. (**E**–**G**) Immediately after stroke onset, mice were injected with a platelet-depleting antibody. One hour later, either recombinant HMGB1 (rHMGB1) or vehicle was administered. Twenty-four hours after stroke induction, plasma MPO-DNA complexes (**E**) were assessed as well as brain infarct volume (**F** and **G**). *n =* 7–9 per group. (**H**–**K**) Twenty-four hours before stroke induction, neutrophils were depleted by i.p. injection of neutrophil-depleting antibodies. Control mice were injected with an IgG control antibody. Twenty-four hours later, plasma HMGB1 (**H**) and MPO-DNA complexes (**I**) were assessed as well as brain infarct volume (**J** and **K**). *n =* 7–8 per group. (**L**–**N**) Twenty-four hours before stroke induction, neutrophils were depleted by i.p. injection of neutrophil-depleting antibodies. One hour after stroke onset, either rHMGB1 or vehicle was administered. Twenty-four hours after stroke induction, plasma MPO-DNA complexes (**L**) were assessed as well as brain infarct volume (**M** and **N**). *n =* 6–7 per group. Groups were compared by ordinary 1-way ANOVA (**A**, **B**, and **D**) or unpaired *t* test. *P < 0.05, **P < 0.01, ***P < 0.001.

In order to study whether platelet HMGB1 drives NET formation in ischemic stroke, we depleted platelets immediately after stroke onset with an anti-GPIb antibody (Figure 5, A–D, and Supplemental Figure 8A). Twenty-four hours after stroke, thrombocytopenic mice had significantly lower levels of circulating HMGB1 compared with mice treated with an IgG control (5.9 ± 2 ng/mL vs. 9.5 ± 2.8 ng/mL, respectively; Figure 5A). Moreover, the reduction of plasma HMGB1 in thrombocytopenic mice was associated with a significant reduction in plasma MPO-DNA complexes 24 hours after stroke (136% ± 53.1% vs. 295.6% ± 128.1%; Figure 5B). As plasma HMGB1 and NET levels were decreased, we next examined whether thrombocytopenic mice were protected from ischemic stroke brain injury. Consistent with a role for platelet-HMGB1-induced NET formation in ischemic stroke, thrombocytopenic mice were protected from brain injury (45.3 ± 20.3 mm^3^ vs. 88.2 ± 35.2 mm^3^; Figure 5, C and D) and had improved neurological function (Supplemental Figure 8, B and C). To further establish a causal role for HMGB1 in mediating NET formation after stroke, we treated thrombocytopenic mice with recombinant HMGB1 (rHMGB1). rHMGB1 increased plasma NET levels in platelet-depleted mice (245.1% ± 81.1% vs. 123.1% ± 35.6%; Figure 5E) and exacerbated ischemic stroke brain injury (56.4 ± 19.3 mm^3^ vs. 28.4 ± 11.2 mm^3^; Figure 5, F and G, and Supplemental Figure 9, A and B).

Besides platelets, neutrophils also express and release HMGB1 (21). To investigate the contribution of neutrophils to the rise of HMGB1 after stroke, we specifically depleted neutrophils before stroke induction (Supplemental Figure 10, A and B, and ref. 22). Twenty-four hours after stroke, neutrophil-depleted mice had similar plasma HMGB1 levels compared with neutrophil-sufficient mice (9.8 ± 3.7 ng/mL vs. 8.8 ± 2.8 ng/mL, respectively; Figure 5H), indicating that neutrophils do not contribute to the rise of plasma HMGB1 after stroke. Nevertheless, neutrophil-depleted mice had reduced levels of circulating MPO-DNA complexes (112.8% ± 46.6% vs. 231.3% ± 74.8%; Figure 5I) and were protected from ischemic stroke brain injury (47.5 ± 15.8 mm^3^ vs. 73 ± 19.5 mm^3^; Figure 5, J and K, and Supplemental Figure 10, C and D). In agreement with our hypothesis, rHMGB1 did not increase plasma NET levels in neutropenic mice (104.7% ± 13.7% vs. 94.7% ± 17.1%; Figure 5L), nor did it alter ischemic stroke brain injury (43.8 ± 19 mm^3^ vs. 47.7 ± 12.5 mm^3^; Figure 5, M and N, and Supplemental Figure 11).

Finally, we treated WT mice with BoxA (20), a specific competitive inhibitor of HMGB1, and measured plasma NET levels and stroke outcomes 24 hours after stroke onset. Treatment with BoxA significantly reduced plasma MPO-DNA complexes (144.8% ± 56.2% vs. 275.4% ± 52.5%; Supplemental Figure 12A) and improved ischemic stroke outcomes (53.9 ± 26.7 mm^3^ vs. 88.2 ± 35.2 mm^3^; Supplemental Figure 12, B–D), confirming a key role for HMGB1 mediating detrimental NET formation in ischemic stroke.

### Platelet-specific HMGB1 knockout reduces platelet-induced NET formation and improves stroke outcomes.

**Figure 6:**
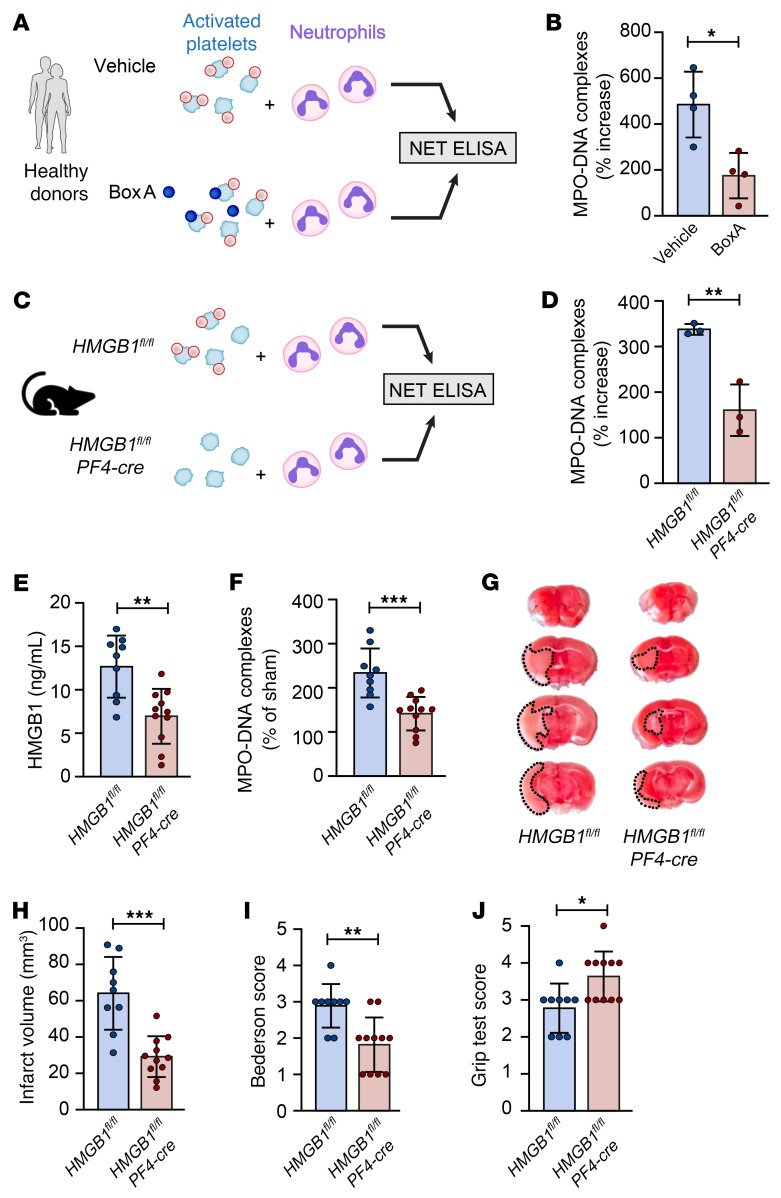
Platelet-specific HMGB1 knockout blocks platelet-induced NET formation and improves stroke outcomes. (**A** and **B**) Platelets and neutrophils were isolated from 4 healthy donors. Platelets were activated for 15 minutes with convulxin and then incubated for 2.5 hours with neutrophils in the presence of BoxA or vehicle, after which NETs were quantified using a MPO-DNA ELISA. *n =* 4 per group. (**C** and **D**) Platelets were isolated from 3 HMGB1^fl/fl^ (WT) or HMGB1^fl/fl^ PF4-cre (KO) mice, activated with convulxin, and incubated for 2.5 hours with WT neutrophils, after which NETs were quantified using an MPO-DNA ELISA. *n =* 3 per group. (**E**–**J**) HMGB1^fl/fl^ (WT; *n =* 9) or HMGB1^fl/fl^ PF4-cre (KO; *n* = 11) mice were subjected to 1 hour of tMCAO followed by 23 hours of reperfusion. Plasma was isolated and brains were analyzed for ischemic stroke brain damage by TTC staining 24 hours after stroke onset. Upon TTC staining, live brain tissue will stain red, while dead brain tissue will remain white (outlined with black dotted line). (**E**) Plasma HMGB1 levels were measured by ELISA. (**F**) Plasma NET levels were measured by MPO-DNA complex ELISA. (**G** and **H**) Infarct size was determined by TTC staining and planimetric analysis. (**I**) Neurological score was measured 24 hours after stroke using Bederson’s test. (**J**) Motor function was assessed 24 hours after stroke using the grip test. Groups were compared by unpaired *t* test (**B**, **D**–**F**, and **H**) or Mann-Whitney test (**I** and **J**). *P < 0.05, **P < 0.01, ***P < 0.001.

To further establish platelet HMGB1 as a critical mediator of NET formation, we isolated platelets and neutrophils from healthy donors and performed in vitro NET assays. Platelets were activated with convulxin (a glycoprotein VI agonist) for 15 minutes and subsequently incubated with neutrophils for 150 minutes, after which NET formation was analyzed by MPO-DNA ELISA. In line with our hypothesis and published literature (20), the competitive HMGB1 inhibitor, BoxA, significantly reduced platelet-induced NET formation (Figure 6, A and B). Since, in this experimental setup, HMGB1 could be released from neutrophils, we performed a similar experiment in mice, using platelets from platelet-specific HMGB1-knockout mice (HMGB1^fl/fl^ PF4-cre; KO) or their littermate controls (HMGB1^fl/fl^; WT) (19). Critically, activated HMGB1-KO platelets failed to induce NETs, resulting in significantly reduced MPO-DNA complexes in comparison with WT littermate controls (Figure 6, C and D).

Subsequently, platelet-specific HMGB1-KO mice and their WT littermate controls were subjected to ischemic stroke, after which plasma was collected and stroke outcomes were analyzed. Twenty-four hours after stroke, HMGB1-KO mice had significantly lower plasma HMGB1 levels compared with WT mice (6.9 ± 3.1 ng/mL vs. 12.7 ± 3.6 ng/mL, respectively; Figure 6E). Importantly, this reduction in plasma HMGB1 was associated with reduced plasma NET levels (141.5% ± 38.1% vs. 233.7% ± 55.5%; Figure 6F) and greatly improved ischemic stroke outcomes (Figure 6, G–J). Together our results confirm a critical role for platelet-derived HMGB1 in the formation of detrimental NETs in the acute phase of ischemic stroke.

### Prophylactic treatment with a NET-inhibitory peptide improves stroke outcomes.

**Figure 7:**
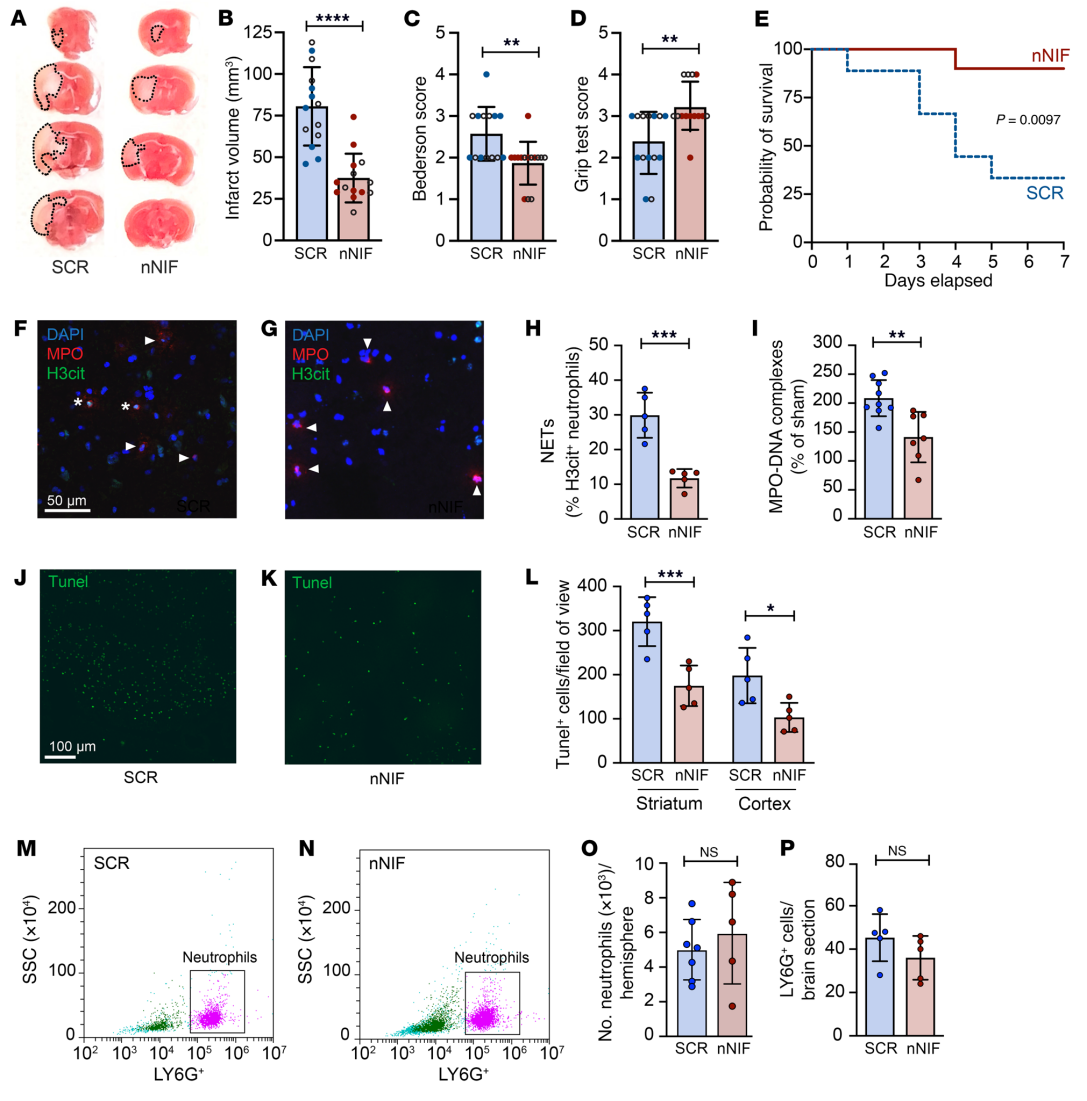
Prophylactic treatment with nNIF protects mice from ischemic stroke brain injury. Mice were subjected to 1 hour of tMCAO followed by 23 hours of reperfusion. Mice were treated with nNIF or SCR 1 hour before and 1 hour after stroke onset (10 mg/kg). Open circles, females; filled circles, males. (**A**) Brain sections were stained with TTC. Red areas indicate healthy brain tissue; white areas show infarcted brain tissue (outlined with black dotted line). (**B**) Quantification of brain infarct volumes 24 hours after stroke. (**C**) Bederson’s test was used to assess neurological outcome 24 hours after stroke. (**D**) Twenty-four hours after stroke, motor function was measured using the grip test. *n =* 14 per group. (**E**) In a separate experiment, mice were followed for up to 7 days after stroke; survival was monitored. *n* = 9 for SCR; *n* = 10 for nNIF. (**F**–**H**) NETs in brain tissue were identified by staining for MPO (red), H3cit (green), and DNA (DAPI; blue). The percentage of NET-forming neutrophils was quantified by counting of H3cit^+^ neutrophils. *n =* 5 per group. Scale bar: 50 μm. (**I**) Plasma NETs were measured 24 hours after stroke using MPO-DNA complex ELISA. *n =* 7–9 per group. (**J**–**L**) Brain sections were stained for apoptosis by labeling of DNA strand breaks with TUNEL (green). TUNEL^+^ cells were counted in the striatum and cortex. *n =* 5 per group. Scale bar: 100 μm. (**M**–**O**) Flow cytometric analysis of single-cell suspensions of ipsilesional brain hemispheres. CD45^+^CD11b^+^Ly6G^+^ cells were counted in nNIF- and SCR-treated animals. *n =* 5–7 per group. (**P**) Quantification of neutrophil staining with Ly6G in brain sections 24 hours after stroke. *n =* 5 per group. Groups were compared by unpaired *t* test (**B**, **H**, **I**, **O**, and **P**), Mann-Whitney test (**C** and **D**), ordinary 1-way ANOVA (**L**), or log-rank test (**E**). *P < 0.05, **P < 0.01, ***P < 0.001, ****P < 0.0001.

HMGB1 has been shown to be important in the resolution of inflammation (23). This limits the clinical use of strategies targeting HMGB1 in ischemic stroke and emphasizes the importance of targeting detrimental downstream effects of HMGB1 signaling. Recently, our laboratory discovered a class of endogenous NET-inhibitory peptides circulating in human umbilical cord blood (24–26). Importantly, NET-inhibitory peptides block NET formation without affecting other neutrophil functions, providing an alternative approach to safely target NET formation and immunothrombosis (25, 26). Based on the sequence of the endogenous NET-inhibitory peptide toward the N-terminus of α_1_-antitrypsin, we synthethized neonatal NET-inhibitory factor (nNIF) (26) and assessed its ability to block platelet-induced NET formation in vitro as well as its therapeutic potential in our ischemic stroke model. In both human and mouse neutrophils, nNIF treatment was effective at preventing platelet-induced NET formation (Supplemental Figure 13). Importantly, when mice were prophylactically treated with nNIF, brain infarct size was reduced significantly in comparison with mice treated with scrambled peptide control (SCR) (37.5 ± 14.6 mm^3^ vs. 80.6 ± 23.5 mm^3^; Figure 7, A and B). Moreover, this protective effect was also evident by significantly improved neurological and motor function 24 hours after stroke onset (Figure 7, C and D). In accordance with the STAIR criteria (27), we used male and female mice in our studies and observed an equal protective effect in both sexes (Figure 7, A–D). In a separate experiment, we pretreated mice with nNIF or SCR and followed mice for 7 days after stroke onset. Treatment with nNIF significantly protected mice from stroke-induced mortality (Figure 7E), suggesting that nNIF improves short- and long-term outcomes following ischemic stroke.

### nNIF prevents neuronal apoptosis by blocking NET formation.

To assess whether nNIF was specifically blocking NET formation, we analyzed brain sections for the presence of NETs by staining for DNA, MPO, and H3cit. nNIF treatment significantly reduced NETs found in ischemic brain tissue after stroke in comparison with SCR-treated animals (11.7% ± 2.7% H3cit^+^ neutrophils vs. 29.9% ± 6.5% H3cit^+^ neutrophils; Figure 7, F–H). Moreover, circulating MPO-DNA complexes were significantly reduced in nNIF-treated mice (141.3% ± 43.7% vs. 208.6% ± 31.3%; Figure 7I). As expected, nNIF treatment did not impact HMGB1 levels after stroke (Supplemental Figure 14). To determine whether reduced NET formation had a functional effect on neuronal cells, we examined neuronal apoptosis after stroke by performing TUNEL staining. In agreement with reduced cerebral infarct sizes, nNIF reduced apoptosis in the ipsilateral brain hemisphere (Figure 7, J–L). Interestingly, nNIF reduced apoptosis in both the infarct core (striatum; 174.8 ± 46.1 cells vs. 320 ± 55.5 cells) and the penumbral cortex (103.2 ± 32.9 cells vs. 198 ± 62.8 cells). Additional staining using NeuN (a neuronal marker; Supplemental Figure 15) confirmed that apoptotic cells in the brain after stroke were of neuronal origin. Next, we quantified brain neutrophil recruitment by flow cytometry of single-cell suspensions of the ipsilesional brain hemisphere. Specific quantification of neutrophil recruitment revealed no difference between nNIF- and SCR-treated animals (5918 ± 2930 Ly6G^+^ neutrophils vs. 5006 ± 1743 Ly6G^+^ neutrophils; Figure 7, M–O). These results were confirmed via immunohistological staining for Ly6G^+^ neutrophils (Figure 7P).

**Figure 8:**
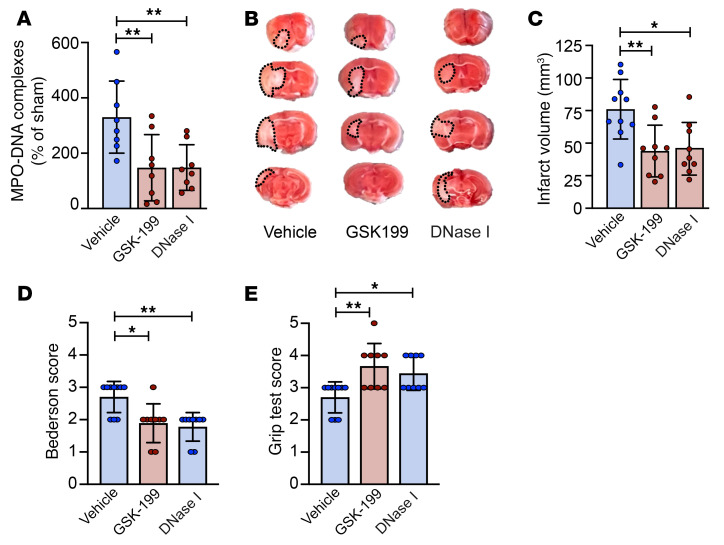
Preventing NET formation or degrading NETs improves ischemic stroke outcomes. Mice were subjected to 1 hour of tMCAO followed by 23 hours of reperfusion. Before ischemic stroke onset, GSK-199 (PAD4 inhibitor to prevent NETs; 30 mg/kg; *n =* 9), DNase I (to degrade NETs; 2.5 mg/kg; *n =* 9), or vehicle (*n =* 10) was injected. (**A**) Plasma NETs were measured 24 hours after stroke using the MPO-DNA complex ELISA. (**B**) Brain sections were stained with TTC. Red areas indicate healthy brain tissue, while white areas show infarcted brain tissue (outlined with black dotted line). (**C**) Quantification of brain infarct volumes 24 hours after stroke. (**D**) Bederson’s test was used to assess neurological outcome 24 hours after stroke. (**E**) Twenty-four hours after stroke, motor function was measured using the grip test. Groups were compared by ordinary 1-way ANOVA (**A** and **C**) or Kruskal-Wallis test (**D** and **E**). *P < 0.05, **P < 0.01.

To confirm a critical role for NETs in mediating ischemic stroke brain injury, we investigated alternative ways of targeting NETs in our ischemic stroke model. PAD4-mediated citrullination of histones is a critical step in the formation of NETs (28), and this process can be inhibited in vivo by GSK-199, a specific PAD4 inhibitor (29, 30). In agreement with the nNIF results, pretreatment of mice with GSK-199 reduced plasma NET levels (147.5% ± 119.5% vs. 330.4% ± 130.3%; Figure 8A) and improved ischemic stroke outcomes as measured by brain infarct size (44.1 ± 19.8 mm^3^ vs. 76.1 ± 22.9 mm^3^; Figure 8, B and C) as well as neurological and motor function (Figure 8, D and E). Similar results were obtained when NETs were degraded with DNase I. DNase I reduced NET levels (148.4% ± 82.5% vs. 330.4% ± 130.3%; Figure 8A) and ischemic brain injury (45.7 ± 20.2 mm^3^ vs. 76.1 ± 22.9 mm^3^; Figure 8, B and C) and improved neurological and motor outcomes (Figure 8, D and E).

### nNIF reduces ischemic stroke brain injury in mice with comorbidities.

**Figure 9:**
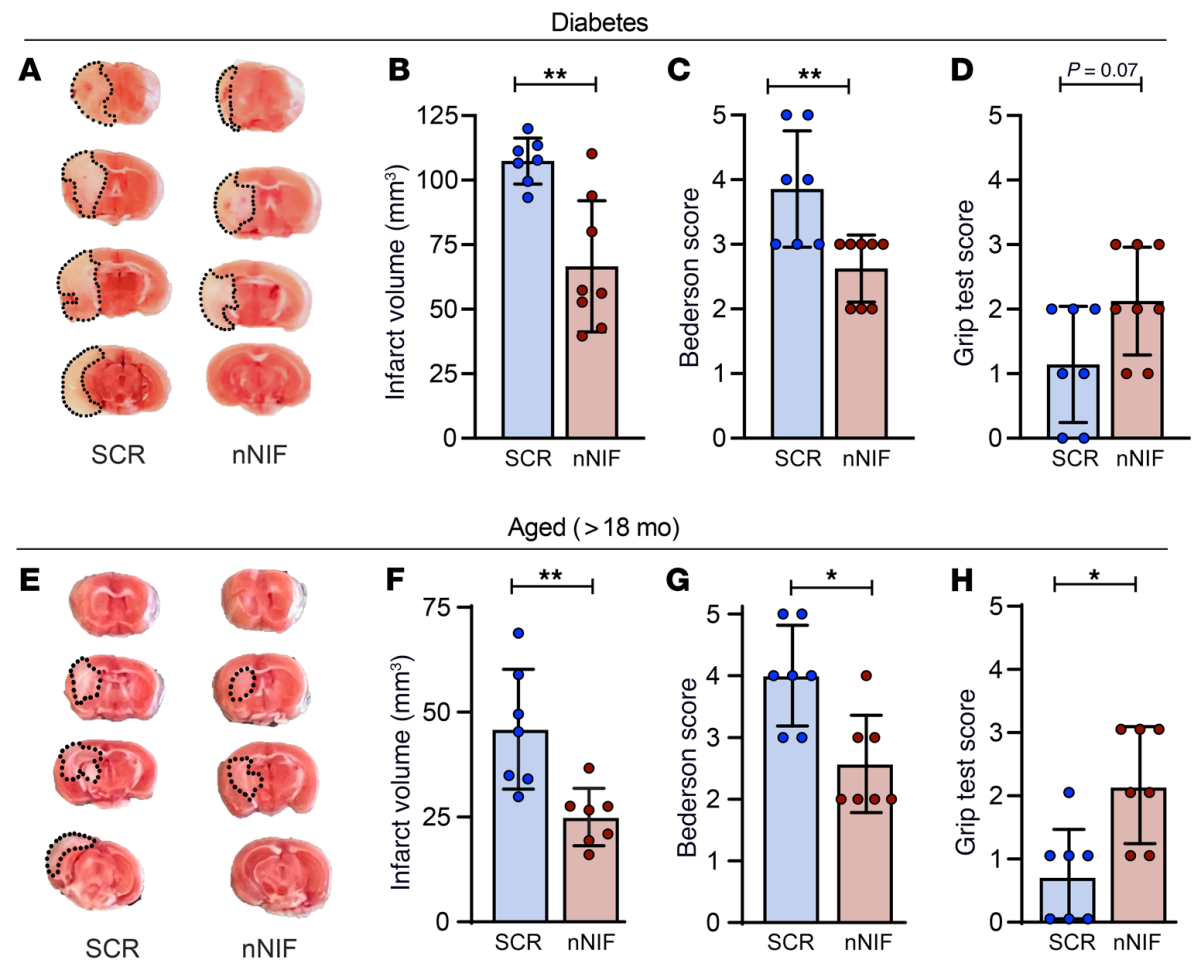
Prophylactic nNIF protects mice with diabetes and aged mice from ischemic stroke brain injury. (**A** and **B**) Diabetic mice were subjected to 1 hour of tMCAO followed by 23 hours of reperfusion. Mice were treated with nNIF or SCR 1 hour before and 1 hour after stroke onset (10 mg/kg). (**A**) Brain sections were stained with TTC. Red areas indicate healthy brain tissue, while white areas show infarcted brain tissue (outlined with black dotted line). (**B**) Quantification of brain infarct volumes 24 hours after stroke. (**C**) Bederson’s test was used to assess neurological outcome 24 hours after stroke. (**D**) Twenty-four hours after stroke, motor function was measured using the grip test. *n =* 7–8 per group. (**E**–**H**) Aged mice (18 months old) were subjected to 1 hour of tMCAO followed by 23 hours of reperfusion. Mice were treated with nNIF or SCR 1 hour before and 1 hour after stroke onset (10 mg/kg). (**E**) Brain sections were stained with TTC. Red areas indicate healthy brain tissue, while white areas show infarcted brain tissue (outlined with black dotted line). (**F**) Quantification of brain infarct volumes 24 hours after stroke. (**G**) Bederson’s test was used to assess neurological outcome 24 hours after stroke. (**H**) Twenty-four hours after stroke, motor function was measured using the grip test. *n =* 7 per group. Groups were compared by unpaired *t* test (**B** and **F**) or Mann-Whitney test (**C**, **D**, **G**, and **H**). *P < 0.05, **P < 0.01.

A common limitation of preclinical stroke studies is the use of young and healthy adult animals. To overcome this limitation, we assessed the effectiveness of nNIF in mice with type 1 diabetes (Figure 9, A–D) and aging (>18 months old; Figure 9, E–H), conditions with an increased stroke risk and aggravated stroke outcomes. Mice were prophylactically treated with nNIF, and 24 hours after stroke, neurological and motor function was assessed before measurement of brain infarction. In diabetic mice, mice treated with nNIF had significantly reduced ischemic stroke brain infarctions (66.6 ± 25.1 mm^3^ vs. 107.4 ± 8.9 mm^3^; Figure 9, A and B). This translated to significantly improved neurological behavior (Figure 9C) and a trend toward improved motor function (Figure 9D). In 18-month-old mice, nNIF significantly reduced brain damage after stroke as measured by infarct volume (25 ± 6.8 mm^3^ vs. 45.9 ± 14.3 mm^3^; Figure 9, E and F) and improved neurological function (Figure 9G) and grip strength (Figure 9H).

### Therapeutic time window of nNIF in murine ischemic stroke.

**Figure 10:**
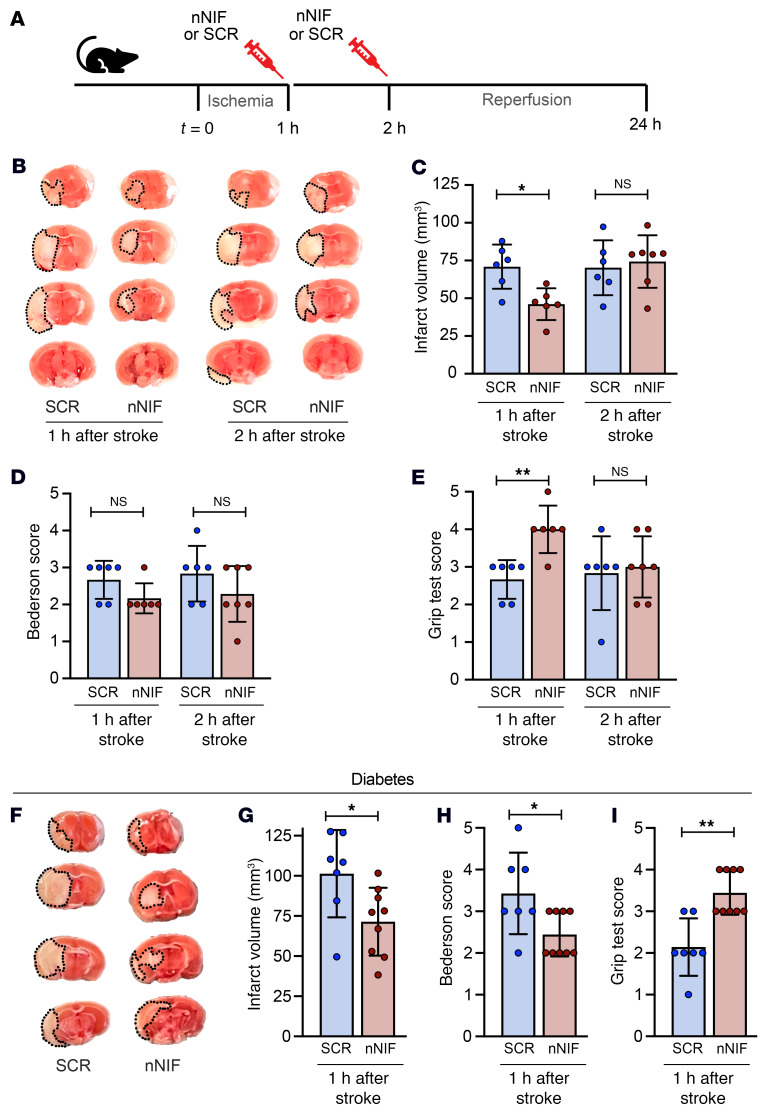
nNIF remains effective when administered up until 1 hour after stroke onset. (**A**–**E**) Mice were subjected to 1 hour of tMCAO followed by 23 hours of reperfusion. (**A**) Mice were treated with nNIF or SCR 1 or 2 hours after stroke onset (10 mg/kg). (**B**) Brain sections were stained with TTC. Red areas indicate healthy brain tissue, while white areas show infarcted brain tissue (outlined with black dotted line). (**C**) Quantification of brain infarct volumes 24 hours after stroke. (**D**) Bederson’s test was used to assess neurological outcome 24 hours after stroke. (**E**) Twenty-four hours after stroke, motor function was measured using the grip test. A higher score indicates better motor function. (**F**–**I**) Diabetic mice were subjected to 1 hour of tMCAO followed by 23 hours of reperfusion. Mice were treated with nNIF or SCR 1 hour after stroke onset. Twenty-four hours later, brain infarct size (**F** and **G**) and neurological (**H**) and motor (**I**) function were assessed. *n =* 6–7 per group. Groups were compared by ordinary 1-way ANOVA (**C**), Kruskal-Wallis test (**D** and **E**), unpaired *t* test (**G**), or Mann-Whitney test (**H** and **I**). *P < 0.05, **P < 0.01.

Next, the therapeutic time window of nNIF in murine ischemic stroke was investigated. For these experiments, nNIF treatment was delayed until 1 or 2 hours after stroke onset, and stroke outcomes were compared with those in SCR-treated mice (Figure 10A). A significant reduction in ischemic stroke brain damage was still observed when nNIF was given 1 hour after stroke onset (46.1 ± 10.5 mm^3^ vs. 70.9 ± 14.6 mm^3^; Figure 10, B and C). However, protection was lost when nNIF was administered 2 hours after stroke onset (74.3 ± 17.4 mm^3^ vs. 70.2 ± 18.2 mm^3^; Figure 10, B and C). Notably, while nNIF treatment 1 hour after stroke had only a minor effect on neurological function (Figure 10D), we did observe a significant improvement in motor function (Figure 10E). Importantly, a similar observation was made in diabetic mice. Treatment with nNIF 1 hour after stroke onset in diabetic mice significantly reduced ischemic brain damage (71.4 ± 21.1 mm^3^ vs. 101.3 ± 27.2 mm^3^; Figure 10, F and G), improved neurological outcomes (Figure 10H), and improved grip strength (Figure 10I).

### Acute NET inhibition confers long-term protection from ischemic stroke.

**Figure 11:**
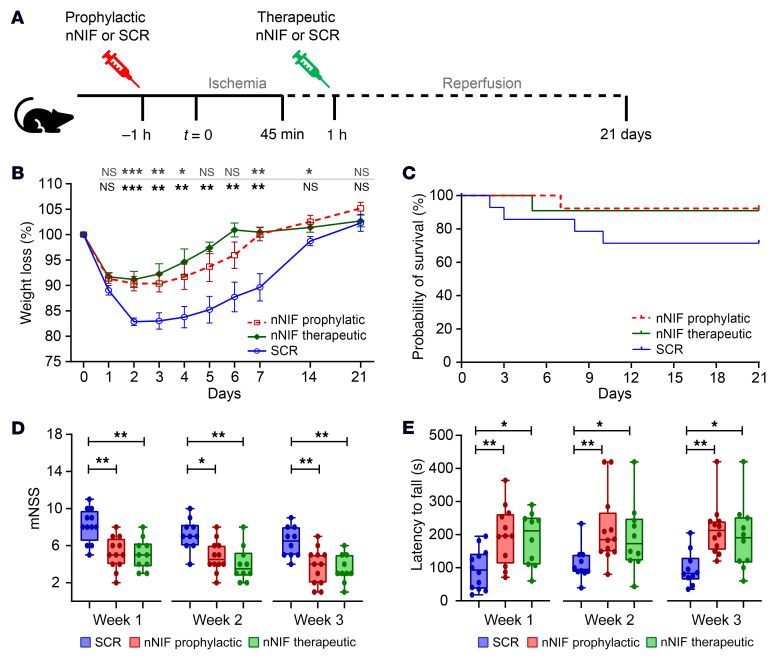
Prophylactic and therapeutic nNIF improves long-term stroke outcomes. Mice were subjected to 45 minutes of tMCAO, after which reperfusion was allowed for 21 days. (**A**) Mice were treated with SCR (10 mg/kg; *n =* 14) or nNIF either 1 hour before (prophylactic; 10 mg/kg; *n =* 13) or 1 hour after stroke onset (therapeutic; 10 mg/kg; *n =* 11). (**B**) Weight was measured every day the first week and then every week. (**C**) Survival was monitored daily for 21 days. (**D**) Modified neurological severity scoring (mNSS) was performed every week after stroke for 3 weeks. (**E**) Motor function was assessed on the accelerated rotarod, and latency to fall was recorded every week after stroke for 3 weeks. Groups were compared by 2-way ANOVA (**B**), log-rank test (**C**), or Kruskal-Wallis test (**D** and **E**). *P < 0.05, **P < 0.01, ***P < 0.001.

Finally, to examine long-term effects of nNIF on stroke outcomes, we subjected mice to 45 minutes of ischemia after which reperfusion was allowed and mice were followed for 3 weeks (Figure 11A). This shorter duration of ischemia was chosen to increase survival rates and have more power to detect potential differences in long-term stroke outcomes. Mice were treated either prophylactically (1 hour before stroke onset) or therapeutically (1 hour after stroke onset) with nNIF or SCR. To monitor overall well-being of mice, weights were measured on a daily basis for the first week after stroke, after which weight was measured every week (Figure 11B). On day 1 after stroke, no differences in weight loss were observed between any of the treatment groups. However, starting from day 2, a clear difference in weight loss became apparent between the nNIF-treated animals and the SCR-treated animals. By 2 weeks after stroke, most mice had recovered to their baseline weight. Also with regard to survival, better outcomes were observed in nNIF-treated animals (Figure 11C). While 28.6% of SCR-treated animals died, only 7.3% of the mice treated with prophylactic nNIF and 9.1% of those treated with therapeutic nNIF succumbed to stroke. However, this difference was not found to be statistically significant (*P =* 0.17).

To assess behavioral and motor stroke outcomes, we performed a modified neurological severity scoring (mNSS) test (31) and assessed motor function using a rotarod (32) every week for 3 weeks after stroke onset. The mNSS scores neurological functioning by analyzing a composite of motor, sensory, reflex, and balancing behavior. At all time points assessed, mice treated with prophylactic or therapeutic nNIF had significantly lower mNSS compared with SCR-treated animals (Figure 11D). In parallel, motor coordination was investigated using the rotarod test. In this test, mice are placed on an accelerating rotarod and the time a mouse remains on the device is recorded. Both prophylactic nNIF– and therapeutic nNIF–treated mice performed significantly better compared with SCR-treated animals (Figure 11E).

## Discussion

In this study, we report the presence of NETs in the brains of ischemic stroke patients. Furthermore, we found increased markers of NET formation in ischemic stroke patients, irrespective of stroke severity and predictive of stroke outcomes. By studying plasma, platelets, and thrombi from ischemic stroke patients, we identified platelet-derived HMGB1 as a potential mediator of platelet-induced NET formation during ischemic stroke. In a mouse model of ischemic stroke, we subsequently confirmed platelets as a critical source of HMGB1 during ischemic stroke brain injury and established that HMGB1-mediated NET formation exacerbates ischemic stroke outcomes. Finally, we established a pathological role for NETs in the acute phase of ischemic stroke. Preventing NET formation using a recently identified neonatal NET-inhibitory peptide (nNIF) decreased ischemic stroke brain injury in healthy mice as well as mice with comorbidities without altering neutrophil trafficking to the brain. Importantly, administration of nNIF was effective up to 1 hour after stroke onset and induced sustained long-term protection from stroke. These results indicate that NET inhibition with nNIF is a potential therapeutic strategy for ischemic stroke.

### NETs are found in stroke brains, and plasma NET levels predict outcomes.

NETs are lattices of extracellular DNA released by neutrophils, meant to trap and kill pathogens (7). In the bloodstream, NETs can induce thrombus formation with the intention to prevent dissemination of infectious agents (33). However, excessive NET formation contributes to cardiovascular diseases by stabilizing blood clots (10), mediating microvascular thrombosis (34), and inducing endothelial cell death (35, 36). Several studies have reported the presence of NETs in ischemic stroke thrombi, where they contribute to tissue-type plasminogen activator (t-PA) resistance (10, 11, 37). Here, we demonstrate using colocalization of NET-specific markers (H3cit, NE, MPO, and DNA) that NETs are present in brain tissue of ischemic stroke patients. This demonstrates that NET formation is clinically relevant during human ischemic stroke. Furthermore, neutrophil infiltration and NET staining in the brains from human stroke patients resembled NET staining in the mouse brain after tMCAO. These results suggest that murine tMCAO is a relevant stroke model with regard to NET formation during stroke.

Previously it was shown that NET levels in stroke thrombi positively correlate with poor stroke outcomes (37). Similarly, we observed that highly specific plasma markers of NETs positively correlated with poor stroke outcomes. In line with our results, surrogate, nonspecific NET markers such as plasma cell-free DNA and nucleosomes have previously been linked to worse outcomes a year after ischemic stroke (12). Additionally, we detected reduced activity of DNase in stroke patient plasma compared with plasma from matched healthy donors. Reduced DNase activity has previously been linked to disease outcomes in systemic lupus erythematosus (38, 39), thrombotic microangiopathies (40, 41), and myocardial infarction (34, 42). DNases are part of the endogenous system to degrade NETs, and they help to maintain tissue integrity during inflammation (43). We hypothesize that during stroke the imbalance between increased NET formation and a reduced NET degradation potential exacerbates stroke outcomes. Overall, these results imply a pathological role for NETs in the acute setting of ischemic stroke, contributing to long-term outcomes. However, more studies are needed to confirm and extend these findings. Moreover, owing to tissue processing techniques such as embedding and fixation, the use of thin tissue slices, and the activity of endogenous DNases, many studies, including ours, have not been able to show robust extracellular NETs. NETs are 3D lattices that are best imaged in their native state because of these limitations. To overcome these limitations, future studies should focus on using intravital microscopy of the brain during ischemic stroke, to visualize acute NET formation.

### Platelet-derived HMGB1 mediates NET formation in stroke.

NETs are not only formed in response to infections, but are frequently observed in pathologies involving sterile inflammation (44). Sterile inflammation occurs in the absence of pathogens and is instead caused by tissue trauma or ischemia. When a thrombus obstructs cerebral blood flow, nutrient-deprived neuronal cells quickly release DAMPs, which recruit immune cells to the ischemic brain (45). In parallel, hypoxia activates endothelial cells, further contributing to the recruitment of immune cells and platelets (46). Intriguingly, experimental studies indicate that platelets mediate ischemic stroke brain injury not through platelet-platelet interactions, but rather through platelet–immune cell interactions (18). Platelets recruit neutrophils directly to the brain by acting as a bridge with the inflamed endothelium. However, activated platelets also release DAMPs, further stimulating inflammation in the ischemic brain.

One critical DAMP expressed and released by platelets is HMGB1 (19). HMGB1 is a DNA-binding protein that has procoagulant and proinflammatory functions and mediates NET formation in venous thrombosis (19, 47), trauma (19), and cancer (48). Plasma levels of HMGB1 are increased in stroke (49, 50) and are even more elevated in the ischemic cerebral compartment in the acute phase of stroke (51). Furthermore, HMGB1 levels predict reperfusion injury in ischemic stroke patients (52). However, the source of HMGB1 as well as the function of HMGB1 in stroke is not well defined. Here, we demonstrate that platelets substantially contribute to the rise in plasma HMGB1 levels in the acute phase of murine ischemic stroke. Furthermore, plasma levels of HMGB1 correlated with plasma NET levels in stroke patients, and platelet-specific knockout of HMGB1 in mice prevented stroke-induced NET formation and improved stroke outcomes. Once released, platelet HMGB1 may activate neutrophils through Toll-like receptor-4 or RAGE, as inhibition of both receptors is protective in thrombosis (19, 53). Nevertheless, the role of HMGB1 in stroke goes beyond its effect on neutrophils. HMGB1 has been shown to be important in the resolution of inflammation (23), and it augments t-PA–induced fibrinolysis and protects against neurotoxicity associated with t-PA (54). These observations limit the clinical use of strategies targeting HMGB1 in ischemic stroke and emphasize the importance of targeting detrimental downstream effects of HMGB1 signaling.

### nNIF protects mice from stroke.

To overcome limitations associated with anti-HMGB1 treatments, we focused on targeting NETs to improve ischemic stroke outcomes. Recently, we discovered that neonatal neutrophils fail to form NETs as a result of circulating NET-inhibitory peptides (NIPs) (24, 26). NIPs are cleavage fragments of α_1_-antitrypsin and specifically block NET formation without affecting other critical neutrophil functions such as chemotaxis or phagocytosis, indicating that NETs are a significant source of injurious inflammation in the setting of ischemic stroke (25, 26). Prophylactic treatment of mice with nNIF induced significant protection from ischemic stroke brain injury, as measured by neurological and motor function, infarct volume, survival, and long-term neurological and motor outcomes. Both circulating and brain NETs were strongly reduced in nNIF-treated animals. Importantly, the reduction in NETs was accompanied by a strong reduction in apoptosis in the affected brain hemisphere. NETs most likely impact stroke outcomes both by amplifying cerebral microthrombosis and by exacerbating neuronal injury, as NETs have previously been shown to be neurotoxic in vitro (55). Our results confirm these findings and extend them to the setting of ischemic stroke.

Similarly to nNIF, blocking of NET formation by PAD4 inhibition or digestion of NETs with DNase I protected mice from ischemic stroke. Chemical inhibitors of PAD4 have been pursued for autoimmune diseases and venous thromboembolism, but clinical development was recently halted for unknown reasons (30). For stroke, inhibition of PAD4 will be challenging because of a high expression of neuronal PAD4, of which the function is currently unknown (56–58). Likewise, DNase I treatment is not specific to NETs (59), and when it is combined with t-PA, bleeding complications have previously been reported (60). Importantly for clinical translation of nNIF, nNIF treatment was equally effective in male and female mice, and protected diabetic and aged mice from ischemic stroke brain injury. Moreover, NET inhibition was effective up to 1 hour after stroke onset. This indicates a critical role for NETs in the acute phase of stroke and implies that NET formation starts early after stroke onset in mice. Whether this happens in a similar time frame in human stroke patients remains to be investigated. However, our observations of similar NET staining in postmortem samples of human and mouse stroke brains suggest that comparable events are happening in human and mouse stroke and hint toward a key role for neutrophils in ischemic stroke pathophysiology. Notably, human blood is very neutrophil rich (50%–70% neutrophils) compared with mouse blood (10%–25% neutrophils), and murine neutrophils do not have defensins that play a role in human neutrophils biology (61). These and other differences in murine and human neutrophil biology warrant caution in extrapolation of our current findings to the clinical stroke setting. Nevertheless, NIPs are an endogenous defense mechanism against excessive NET formation, conserved across species (25, 26), and therefore hold great therapeutic potential.

It will be important for future studies to use specific NET markers to validate our clinical study in a bigger cohort, as a limitation of our study was a moderately small patient sample size. In addition, future studies are needed to examine the time course of NET formation in stroke patients to identify a time window when nNIF is most effective. Finally, studies will need to examine the protective effect of nNIF in large-animal models of stroke before piloting of the utility of nNIF in human stroke.

### Conclusions.

We established that platelets induce detrimental NET formation during ischemic stroke through an HMGB1-dependent mechanism. Specifically targeting NETs using nNIF protected both healthy mice and mice with comorbidities from ischemic stroke. Combined, our results highlight that targeting NETs is a promising therapeutic strategy for ischemic stroke.

## Methods

### Ischemic stroke patients and samples

All subjects provided written, informed consent, and all study protocols were IRB approved. Ischemic stroke patients (*n =* 27) were recruited from the Neuro Critical and Neuro Acute Care Units at the University of Utah Health Sciences Center in Salt Lake City, Utah, between June 2018 and June 2021. Ischemic stroke was verified by imaging in all patients with the exclusion criteria of the presence of a hemorrhagic stroke, the absence of an ischemic stroke, or if it was unclear the patient had an ischemic stroke. The use of t-PA and removal of the thrombus were not exclusion criteria. The prescription of antiplatelet agents (aspirin at any dose, clopidogrel, or NSAIDs) was recorded based on medication reconciliations done by medical staff upon admission. All patients were enrolled within 48 hours of hospital admission and were recruited under protocols approved by the IRB of the University of Utah (IRB 00122895). Healthy age- and sex-matched donors (*n =* 27) were enrolled under a separate protocol (IRB 0051506). Healthy donors had no known bleeding disorder, liver or kidney disease, cancer, or history of surgery or thrombotic event within the past 3 months, and were not on antiplatelet or anticoagulant therapy. Whole blood was collected from stroke patients and healthy donors into acid-citrate-dextrose (3.2%) sterile tubes, and cell-free plasma was isolated as previously described (62). Clinical characteristics of the healthy donors and stroke patients are summarized in Supplemental Table 2.

### Histological processing and staining of ischemic stroke patient brain samples and clots

We obtained brain specimens from the NIH NeuroBioBank from patients who died after ischemic stroke. Specimens were from the right superior temporal lobe in Brodmann’s area 22 with contralateral tissue from the same area as control. All 3 patients were male, with an average age of 81. Samples were collected after written or telephone consent of the next of kin within 24 hours postmortem for 2 samples and 48 hours for 1 sample. Two patients had cerebral infarcts of an unknown cerebral artery, and a third patient had cerebral infarct in the right middle cerebral artery. Samples were paraffin embedded and processed as described previously (63). Ischemic stroke thrombi were collected from patients undergoing thrombectomy under a protocol approved by the University of Utah IRB (IRB 00132234; no informed consent was required). Immediately after thrombectomy, clot specimens were washed in 0.9% saline and fixed overnight in 4% paraformaldehyde. After a sucrose gradient, clot specimens were snap-frozen in OCT compound and stored at –80°C until cryosectioning into 10 μm slices (64). Before staining, slides were fixed in 4% paraformaldehyde and blocked in 3% donkey serum with 0.5% Tween-20. As primary antibodies, we used goat anti-MPO (2 μg/mL; AF3667, R&D Systems), rabbit anti–human H3cit (2 μg/mL; ab5103, Abcam), mouse anti-hELA2 (2 μg/mL; MAB91671, R&D Systems), mouse anti-GPIb (2 μg/mL; MA5-11642, Invitrogen), and/or rabbit anti-HMGB1 (2 μg/mL; ab18256, Abcam). As secondary antibodies, we used donkey anti–rabbit AF488 (2 μg/mL; R37118, Thermo Fisher Scientific), donkey anti–goat AF546 (2 μg/mL; A-11056, Thermo Fisher Scientific), and donkey anti–mouse AF647 (2 μg/mL; A-31571, Thermo Fisher Scientific). DAPI was used as a nuclear counterstain (300 nM; D1306, Life Technologies). Images were acquired using a high-resolution, confocal reflection microscope (Olympus IX81, FV300).

### ELISAs

#### D-dimer.

Plasma samples were diluted 1:100, and D-dimer was measured following the manufacturer instructions (ab260076, Abcam).

#### Platelet factor 4.

Plasma samples were diluted 1:200, and PF4 was measured following the manufacturer instructions (ab189573, Abcam).

#### Calprotectin.

Plasma samples were diluted 1:100, and calprotectin levels were measured with the Human S100A8/S100A9 Heterodimer DuoSet ELISA according to the manufacturer instructions (DY8226-05, R&D Systems).

#### H3cit.

Plasma samples were diluted 1:2, and H3cit levels were measured using the Citrullinated Histone H3 ELISA Kit according to the manufacturer instructions (501620, Cayman Chemical).

#### MPO-DNA complexes.

An in-house ELISA was used to quantify MPO-DNA complexes. Briefly, after overnight coating with anti-MPO antibody (2 μg/mL; 0400-0002, Bio-Rad) at 4°C, a 96-well plate was blocked with 2.5% BSA in PBS for 2 hours at room temperature. The plate was subsequently washed before incubation for 90 minutes at room temperature with 20% human or mouse plasma in blocking buffer. The plate was washed 5 times, and then incubated for 90 minutes at room temperature with anti-DNA antibody (1:10; Cell Death Detection ELISA, 11544675001, Sigma-Aldrich). After 5 washes, the plate was developed with ABTS substrate (10102946001, Sigma-Aldrich).

#### DNase activity.

Plasma was diluted 1:2.5 and analyzed using a DNase I assay kit according to the manufacturer instructions (ab234056, Abcam).

#### HMGB1.

Human and mouse plasma samples were diluted 1:4, and HMGB1 levels were measured using the HMGB1 ELISA Kit according to the manufacturer instructions (ST51011, Tecan).

### Flow cytometric platelet and neutrophil characterization

To measure platelet-neutrophil aggregates, we diluted whole blood 1:10 with Medium 199 (12-117F, Lonza) containing 100 U/mL heparin. Neutrophils were labeled with V450 anti–human CD66b antibodies (561649, BD Biosciences), and platelets were labeled with APC–anti–human CD41 (559777, BD Biosciences) for 15 minutes at 37°C (63, 65). Samples were fixed with FACS lysis buffer, centrifuged at 500*g* for 10 minutes, and resuspended in PBS before analysis. To detect HMGB1^+^ platelets, washed platelets were resuspended in Medium 199 containing calcium and stained for 15 minutes in the presence of AF488-labeled anti-HMGB1 (ab195010, Abcam) and APC-labeled anti–human CD41. Samples were fixed with FACS lysis buffer and run on a Beckman Coulter Cytoflex located in the Utah Flow Cytometry Core.

### In vitro platelet-induced NET formation

Neutrophils were isolated from freshly collected whole blood of healthy adults or adult mice using the EasySep Direct Human Neutrophil Isolation kit (19666, Stemcell Technologies) or EasySep Mouse Neutrophil Enrichment Kit (19762, Stemcell Technologies), respectively, with greater than 95% purity (63). Neutrophils were resuspended to a concentration of 1 × 10^6^ cells/mL in Medium 199. Platelets were purified as described previously (66, 67), resuspended to 1 × 10^8^ cells/mL in Medium 199, and activated with 50 ng/mL convulxin (sc-202554, Santa Cruz Biotechnology) for 15 minutes. Activated platelets and neutrophils were incubated at a 100:1 ratio to induce NETs for 2.5 hours at 37°C in 5% CO_2_/95% air. HMGB1 was blocked by 10 μg/mL BoxA (HM-014, HMGBiotech). Neutrophils were pretreated for 1 hour with either nNIF or SCR peptides. NET levels were measured using the aforementioned MPO-DNA ELISA.

### Murine model of ischemic stroke

All animal experiments complied with the regulatory standards of the University of Utah (IACUC 21-09012) and were performed following the ARRIVE guidelines (www.nc3rs.org.uk), including randomization and analysis blind to the genotype. All experiments were performed using 10- to 12-week-old male and female C57BL/6J mice (The Jackson Laboratory) except for experiments in which 18-month-old male C57BL/6J mice were used. For these studies, C57BL/6J mice were purchased from The Jackson Laboratory and aged in-house. To study the role of platelet HMGB1, male and female HMGB1^fl/fl^ and HMGB1^fl/fl^ PF4-cre mice on a C57BL/6J background were used (19, 47).

#### Transient middle cerebral artery occlusion stroke model.

Transient middle cerebral artery occlusion (tMCAO) was performed as described previously (67, 68). Briefly, occlusion of the right MCA was achieved by insertion of a standardized monofilament (Doccol Corp.) via the right internal carotid artery to occlude the origin of the right MCA. The occluding suture was left in situ for 60 minutes. Induction of ischemic stroke was confirmed by neurological testing of the mice while the MCA was occluded. Anesthesia was induced by inhalation of 5% isoflurane and maintained by inhalation of 2% isoflurane. Buprenorphine was administered 1 hour before surgery and every 12 hours as needed. Sham surgery was performed similarly, without insertion of the monofilament. The following conditions excluded mice from endpoint analyses (exclusion criteria): (a) death within 12 hours after tMCAO, (b) operation time greater than 10 minutes, or (c) occurrence of surgical complications. Brains of dead mice were visually checked for surgical complications and stained with 2,3,5-triphenyl-tetrazolium chloride (TTC) when possible, as described below, to confirm ischemic stroke–related mortality.

#### Induction of diabetes.

Diabetes was induced as described previously (67). In brief, 50 mg/kg streptozotocin (S0130, Sigma-Aldrich) was administered by i.p. injections for 5 consecutive days at 6 weeks of age. Mice were housed for an additional 2 weeks before being used in experiments. Nonfasting blood glucose concentrations were determined by tail-clip blood draw with a Contour clinical glucometer (Bayer). Only mice with nonfasting blood glucose exceeding 300 mg/dL were used for experiments.

#### Platelet depletion, neutrophil depletion, and drug administration.

Platelets were depleted by i.v. injection of 2 mg/kg platelet-depleting antibodies (R300, Emfret) immediately after stroke induction. Platelet counts were measured using a Hemavet hematology analyzer (Drew Scientific). Neutrophils were depleted by i.p. injection of 5 mg/kg anti–mouse Ly6G (1A8, BE0074-1, Bio X Cell) and 5 mg/kg anti–rat Kappa IgG (MAR 18.5, BE0122, Bio X Cell) (22), 24 hours before stroke induction. Neutrophil depletion was confirmed via flow cytometry using granularity and Ly6C staining (Supplemental Figure 10 and ref. 22). Recombinant disulfide HMGB1 (rHMGB1; HM-120, HMGBiotech) was administered at 0.5 mg/kg i.v. 1 hour after stroke onset. HMGB1 was blocked by injection of 15 mg/kg of BoxA (HM-014, HMGBiotech) immediately before stroke induction. Neonatal NET-inhibitory factor (nNIF) and its inactive, scrambled peptide control (SCR) were synthesized as previously described by the University of Utah DNA/Peptide Synthesis Core Facility and injected i.v. at a concentration of 10 mg/kg at the indicated time points. PAD4 was inhibited by i.v. injection of GSK-199 (17489, Cayman Chemical) at 30 mg/kg, and NETs were degraded by i.v. injection of DNase I (dornase alfa, University of Utah Pharmacy) at 2.5 mg/kg, immediately before stroke induction.

#### Neurological and motor scoring.

Twenty-four hours after stroke onset, mice were subjected to the modified Bederson’s test and the grip test to assess neurological and motor function, respectively (67, 68). To assess long-term neurological outcomes, modified neurological severity scoring (mNSS) was performed every week for 21 days (31). Analysis of long-term motor outcomes was done on an accelerated rotarod (32). The speed of the rod increases with time (start: 2.5 rpm; final: 30 rpm), and the amount of time the animal remains on the device is recorded.

#### Determination of brain infarct size.

To quantify ischemic stroke brain damage, 2-mm-thick coronal brain sections were stained with 2% 2,3,5-triphenyl-tetrazolium chloride (TTC; T8877, Sigma-Aldrich) to distinguish unaffected brain tissue from infarcted tissue, 24 hours after stroke induction (67, 68). Stained slices were photographed, and infarct areas (white) were measured using ImageJ software (NIH) by an operator blinded to treatment.

#### Histology of murine ischemic stroke brains.

Twenty-four hours after stroke, mice were euthanized, and the brains were dissected, snap-frozen in OCT compound, and stored at –80°C until cryosectioning into 10 μm slices. Before immunohistology staining, slides were fixed in 4% paraformaldehyde and blocked in 3% donkey serum with 0.5% Tween-20. As primary antibodies, we used goat anti-MPO (2 μg/mL; AF3667, R&D Systems), rabbit anti–human H3cit (2 μg/mL; ab5103, Abcam), and rat anti-GPIX (2 μg/mL; M051-0, Emfret). For quantification of neutrophils via histology, sections were stained for the presence of Ly6G (rat anti–mouse Ly6G, 2 μg/mL; 127602, BioLegend). As secondary antibodies, we used donkey anti–rabbit AF488 (2 μg/mL; R37118, Thermo Fisher Scientific), donkey anti–goat AF546 (2 μg/mL; A-11056, Thermo Fisher Scientific), donkey anti–rat AF555 (2 μg/mL; A48270, Thermo Fisher Scientific), and donkey anti–mouse AF647 (2 μg/mL; A-31571, Thermo Fisher Scientific) when appropriate. DAPI was used as a nuclear counterstain (300 nM; D1306, Life Technologies). TUNEL staining was performed according to instructions of the In Situ Cell Death Detection Kit (11684795910, Sigma-Aldrich). Images were acquired using a high-resolution, confocal reflection microscope (Olympus IX81, FV300).

#### Mouse stroke brain single-cell flow cytometry.

Twenty-four hours after stroke, mice were euthanized, and single-cell suspensions were made of the ischemic (ipsilesional) hemispheres as previously described (68). Cells were incubated with CD45 APC-Cy7 (103116, BioLegend), CD11b PE-Cy7 (25-0112-82, eBioscience), Ly6G BV510 (127633, BioLegend), and CD16/CD32 (14-0161-82, Fc block, eBioscience) for 30 minutes at room temperature in PBS plus 5% FBS. Thirty minutes later, cells were washed, fixed, and run on a Beckman Coulter Cytoflex located in the Utah Flow Cytometry Core.

### Statistics

Statistical analyses were performed with GraphPad Prism version 9.1.2 (GraphPad Software). Before statistical analysis, D’Agostino and Pearson’s normality test was used to check data distribution. An unpaired, 2-tailed *t* test or 1-way ANOVA with Dunnett’s post hoc test or a Mann-Whitney test, as appropriate, was used for statistical comparison when applicable. In the case of nonparametric data, a Kruskal-Wallis test with post hoc Dunn’s correction was performed. Spearman correlation analysis was performed for all correlation studies. A log-rank (Mantel-Cox) test was used to compare survival. All data are represented as dot plots including a bar graph with error bars representing mean ± SD. A 2-tailed *P* less than 0.05 was considered statistically significant.

### Study approval

All study participants or their legal authorized representative gave written informed consent for study enrollment in accordance with the Declaration of Helsinki. All human and animal studies were approved by the University of Utah IRB or IACUC, respectively.

## Author contributions

FD, IP, JLR, MJC, CvDA, CH, MDA, RG, MRD, MDN, JJM, CCY, and RAC designed and performed experiments. FD and RAC analyzed results, made the figures, and wrote the manuscript. All authors reviewed and critically edited the manuscript.

## Supplementary Material

Supplemental data

Supplemental video 1

## Version 1 03/31/2022

In-Press Preview

## Version 2 05/16/2022

Electronic publication
