# Supplementary material for: Neutrophil extracellular traps regulate ischemic stroke brain injury
Source: J Clin Invest. 2022 May 16;132(10):e154225. doi: 10.1172/JCI154225 (PMC9106355; doi:10.1172/JCI154225)
Supplement: Supplemental data [file jci-132-154225-s270.pdf]

Neutrophil Extracellular Traps Regulate Ischemic Stroke Brain Injury

Frederik Denorme[1]; Irina Portier[1]; John L. Rustad[1]; Mark J. Cody[1]; Claudia V. De Araujo[1]; Chieko Hoki[1]; Matthew D. Alexander[2,3]; Ramesh Grandhi[3,4]; Mitchell R. Dyer[5], Matthew D. Neal[5]; Jennifer J. Majersik[2]; Christian C. Yost[1,6]; and Robert A. Campbell[1,7].

[1]University of Utah Molecular Medicine Program, Salt Lake City, Utah.

[2]Department of Neurology, University of Utah, Salt Lake City, Utah.

[3]Deparment of Radiology and Imaging Sciences, University of Utah, Salt Lake City, Utah

[4]Department of Neurosurgery, University of Utah, Salt Lake City, Utah

[5]Department of Surgery, University of Pittsburgh, Pittsburgh, Pennsylvania.

[6]Department of Pediatrics, University of Utah, Salt Lake City, Utah.

[7]Department of Internal Medicine, University of Utah, Salt Lake City, Utah

Corresponding author:

Robert A. Campbell

University of Utah Health Sciences Center

Eccles Institute of Human Genetics

15 North 2030 East, Bldg. 533, Suite 4225

Salt Lake City, Utah 84112

(801) 585-0950 (phone)

(801) 585-0701 (fax)

rcampbell@u2m2.utah.edu

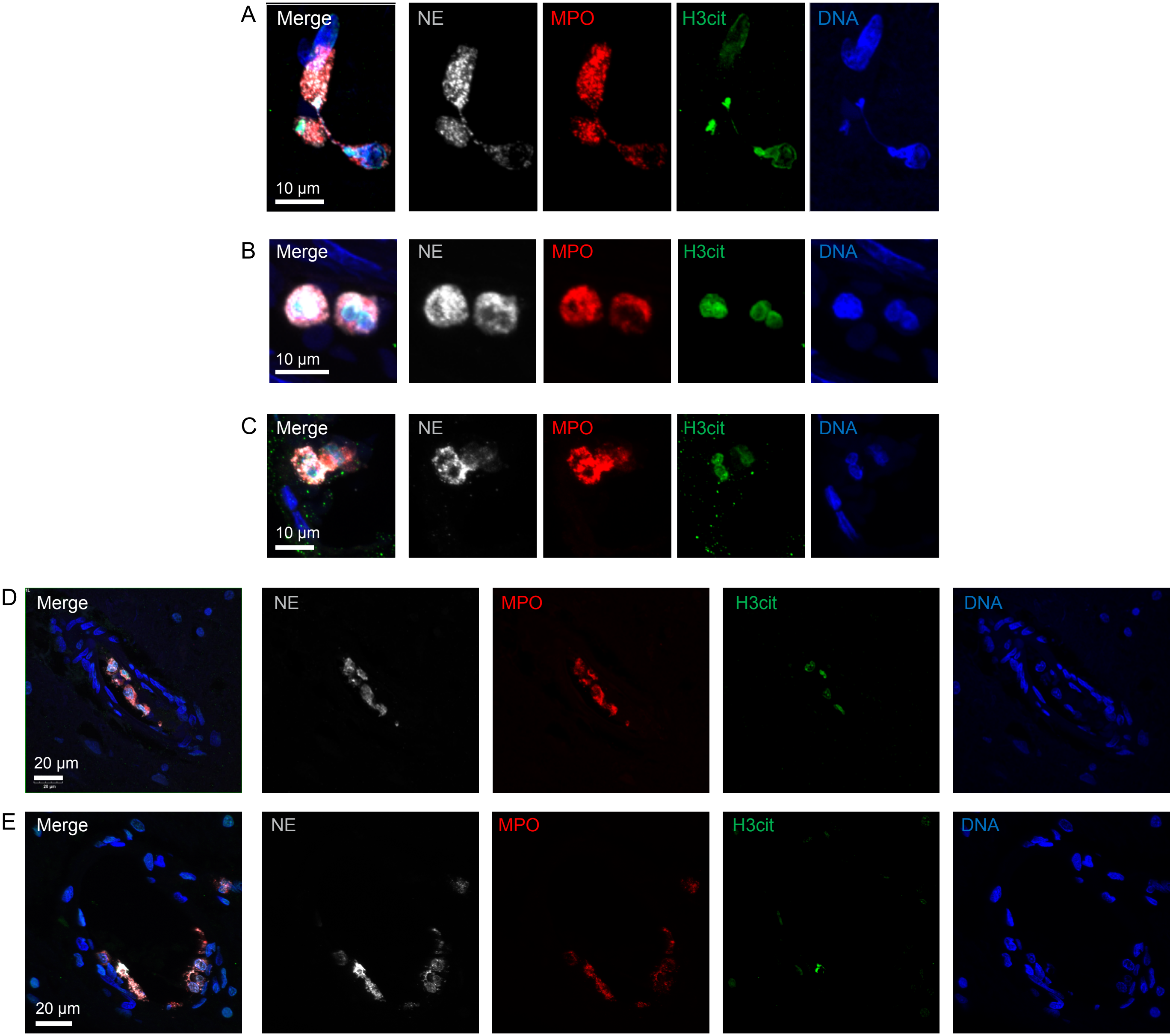

Supplemental Figure 1. Neutrophil Extracellular Traps (NETs) are found in ipsilesional brain tissue from ischemic stroke patients. Ipsilesional brain tissue was obtained from the National Institute of Health Neurobiobank from three patients that died after ischemic stroke. NETs were identified by co-localization of myeloperoxidase (MPO; red), neutrophil elastase (NE; white), citrullinated histone H3 (H3cit; green) and DNA (blue). NET forming neutrophils (A) Patient 1, (B and C) Patient 2, and (D and E) Patient 3. Images shown are representative of a total of three patients.

Contralesional Brain Tissue

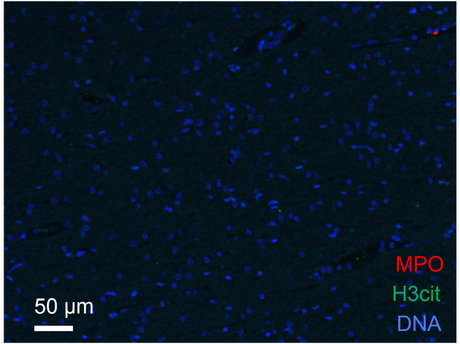

Supplemental Figure 2. Staining for NETs in contralesional brain tissue from ischemic stroke patients. Contralesional brain tissue from the same patient in Figure 1 and Supplemental Figure 1A, was processed and stained for NETs. NETs were stained for using co-localization of myeloperoxidase (MPO; red), citrullinated histone H3 (H3cit; green) and DNA (blue). Image shown is representative of a total of three patients.

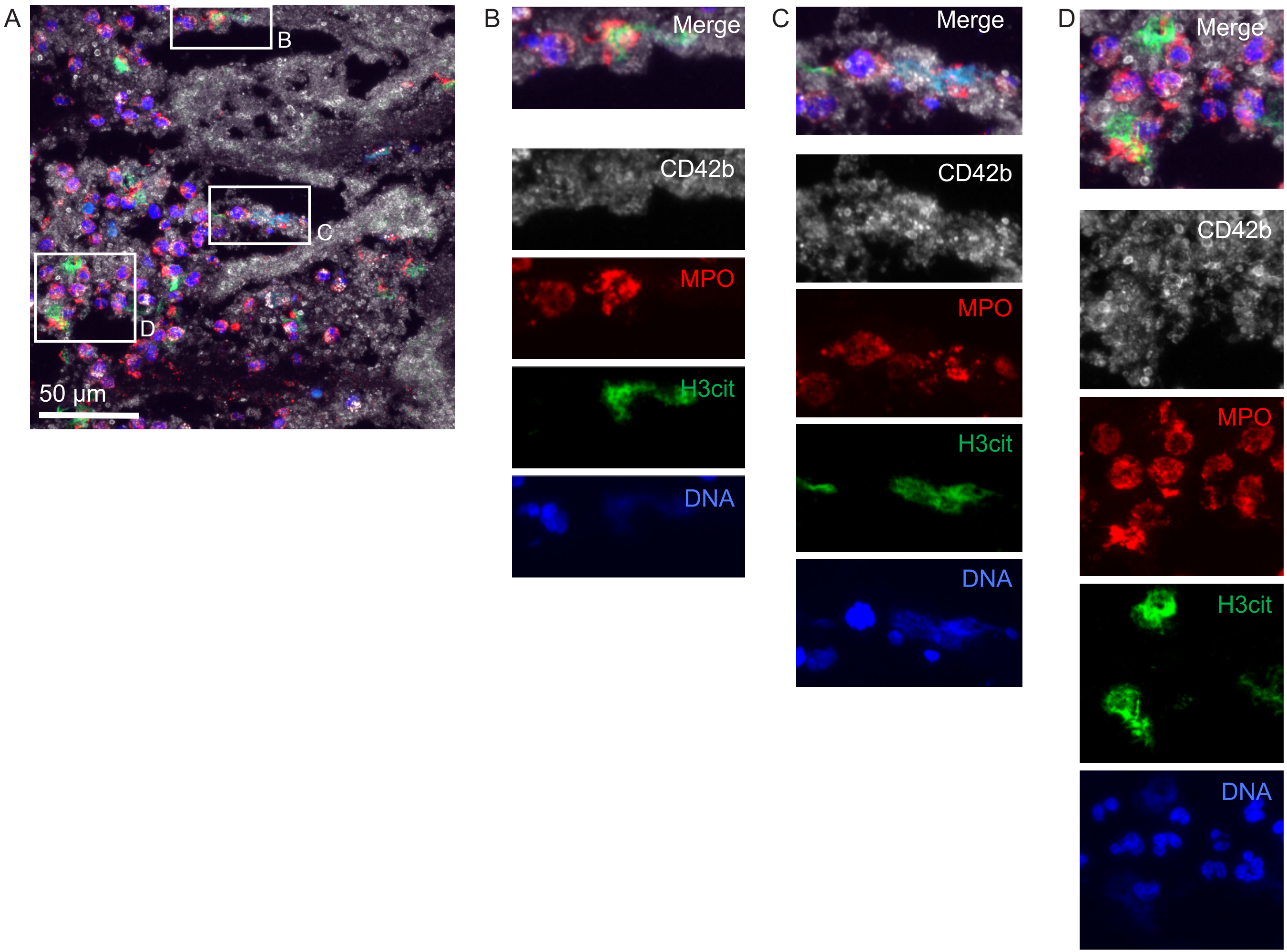

Supplemental Figure 3. Neutrophil Extracellular Traps (NETs) are found in platelet-rich areas in thrombi extracted from ischemic stroke patients. (A) Ischemic stroke thrombi were collected from patients undergoing thrombectomy at the University of Utah. Immediately after thrombectomy, clot specimens were washed, fixed and snap-frozen. Ten µm slices were stained for MPO (red), citrullinated Histone H3 (H3cit; green) and platelets (CD42b; white). DAPI was used as a nuclear counterstain (DNA; blue). Boxes (B, C and D) show higher magnification of NETs in platelet rich areas. Image is representative of 7 ischemic stroke thrombi.

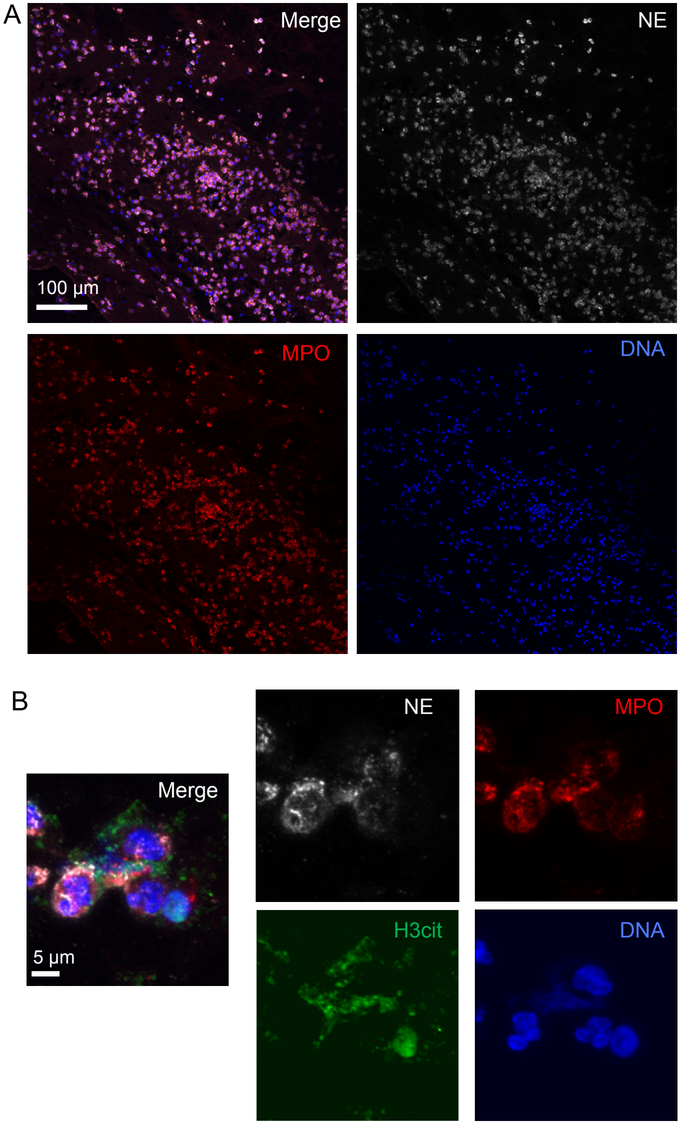

Supplemental Figure 4. Neutrophil elastase and myeloperoxidase colocalize in ischemic stroke thrombi. Ischemic stroke thrombi were collected from patients undergoing thrombectomy at the University of Utah. Immediately after thrombectomy, clot specimens were washed, fixed and snap-frozen. (A) Slides were stained for MPO (red) and NE (white). DAPI was used as a nuclear counterstain (DNA; blue). Low power magnification. (B) Slides were stained for MPO (red), citrullinated Histone H3 (H3cit; green) and NE (white). DAPI was used as a nuclear counterstain (blue). High power magnification. Image is representative of 7 ischemic stroke thrombi.

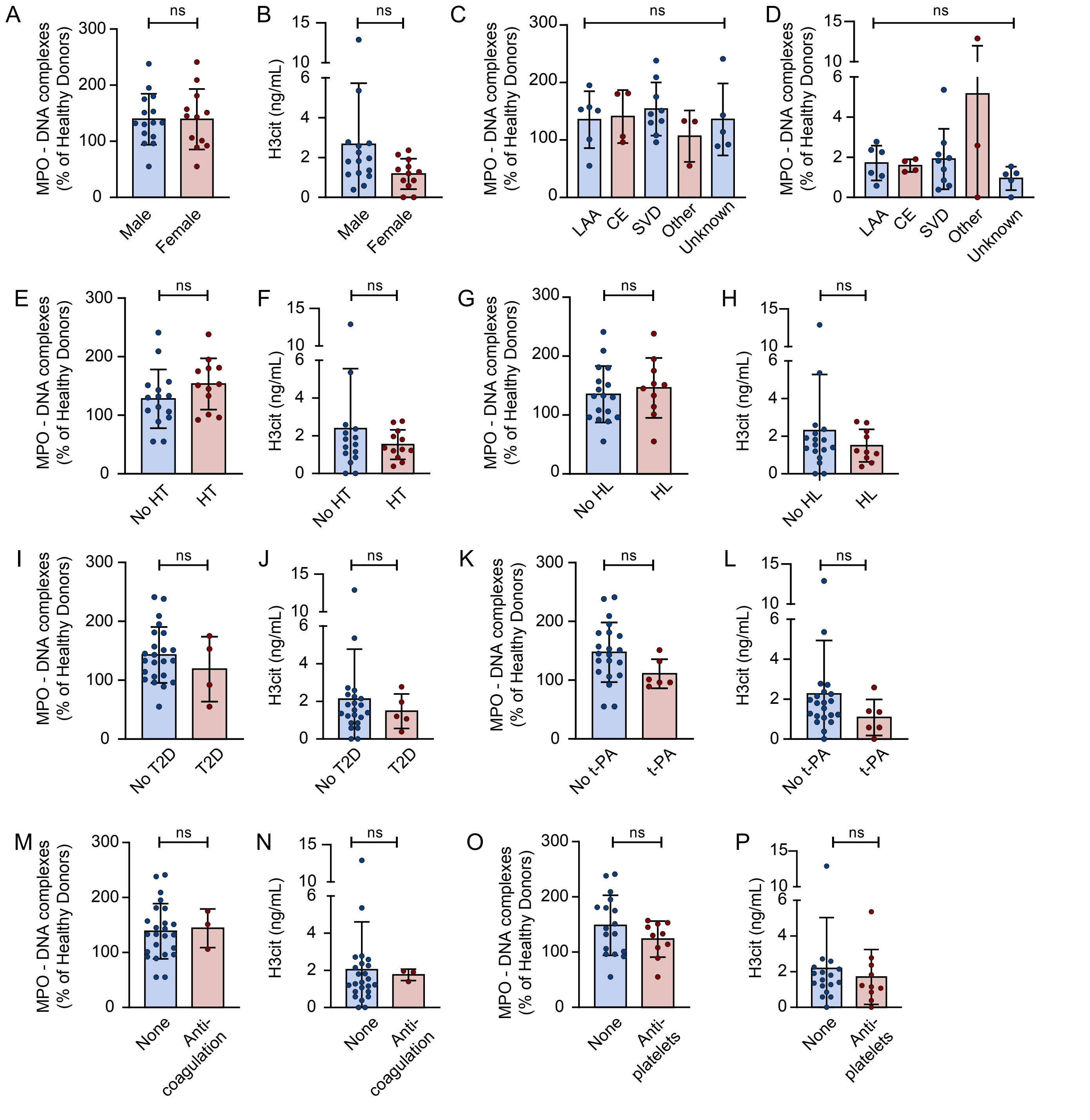

Supplemental Figure 5. Subgroup analysis of NET biomarkers in ischemic stroke patients. Plasma samples were obtained within 48 hours of hospital admission. Stroke patients were divided in groups according to sex (A-B), stroke etiology (C-D), comorbidity (E-J) or medication (K-P). LAA = large artery atherosclerosis, CE = cardioembolic, SVD = small vessel disease. HT = hypertension, HL = hyperlipidemia, T2D = type 2 diabetes. t-PA = tissue plasminogen activator. Anticoagulant was defined by warfarin. Antiplatelets were defined by aspirin or clopidogrel. Data was analyzed using a Mann Whitney test or Kruskal Wallis test where appropriate. N=27. ns = not significantly different.

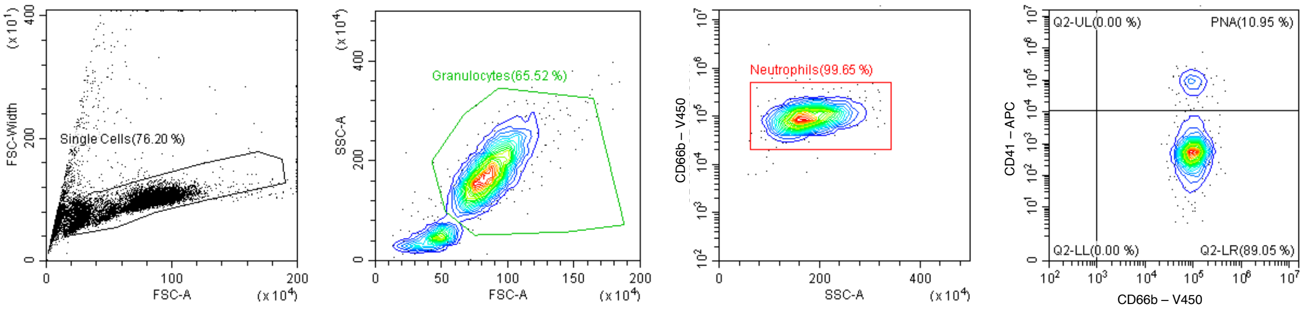

Supplemental Figure 6. Gating strategy for flow cytometric detection of platelet-neutrophil aggregates. Flow cytometry was performed on diluted whole blood isolated from ischemic stroke patients and matched healthy donors to quantify platelet-neutrophil aggregates (PNA). PNA were defined by gating on single cells (A), high granularity (B), CD66b positivity (C) and double staining for CD66b and CD41 (D). Flow plots are representative for N=11-12 per group. Shown is a representative flow plot from an ischemic stroke patient.

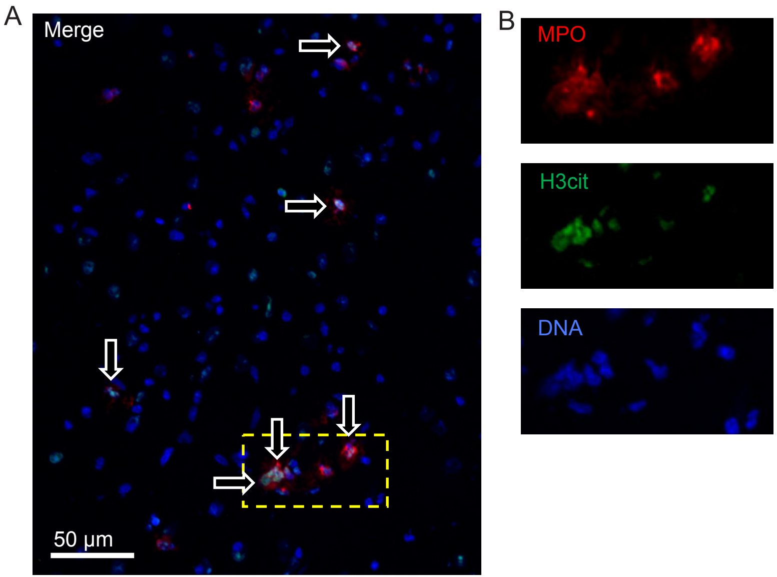

Supplemental Figure 7. Characterization of NET formation in a mouse model of ischemic stroke. (A) Mice were subjected to 1 hour of transient middle cerebral artery occlusion. Brains were collected and processed for histology 24 hours after stroke. Brain tissue was stained for the presence of NETs by a combination of MPO (red), H3cit (green) and DNA (DAPI, blue). Arrows indicate NETting neutrophils. Low power magnification. Box indicates higher magnification shown in (B). Images are representative of 4 mice.

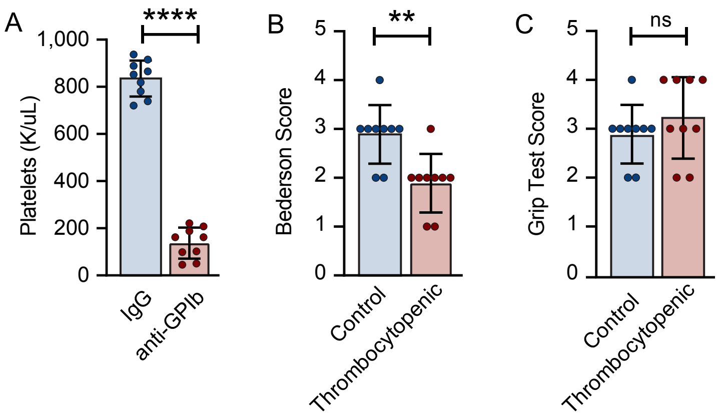

Supplemental Figure 8. Thrombocytopenia improves neurological but not motor outcomes after stroke. Mice were subjected to 1 hour of transient middle cerebral artery occlusion followed by 23 hours of reperfusion. Immediately after stroke onset, mice were injected with a platelet-depleting antibody or IgG control. (A) Platelet counts were measured 23 hours after stroke onset. (B) The Bederson test was used to assess neurological outcome 24 hours after stroke. (C) In parallel, motor function was measured using the grip test. Data was compared between groups using an unpaired t-test (A) or Mann Whitney test (B and C). N=9 per group. **P < 0.01; **** P < 0.0001. Ns = not significantly different.

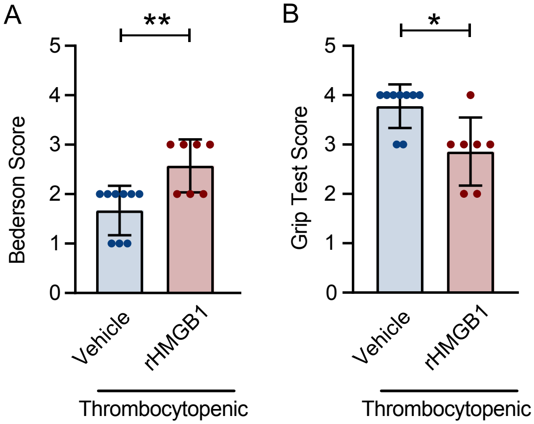

Supplemental Figure 9. Recombinant HMGB1 exacerbates stroke outcomes in thrombocytopenic mice. Mice were subjected to 1 hour of transient middle cerebral artery occlusion followed by 23 hours of reperfusion. Immediately after ischemic stroke onset, platelets were depleted and 1 hour later mice were treated with either recombinant HMGB1 (rHMGB1) or vehicle. (A) The Bederson test was used to assess neurological outcome 24 hours after stroke. (B) In parallel, motor function was measured using the grip test. Data was compared between groups using a Mann Whitney test. N=7-9 per group. *P < 0.05 and **P < 0.01.

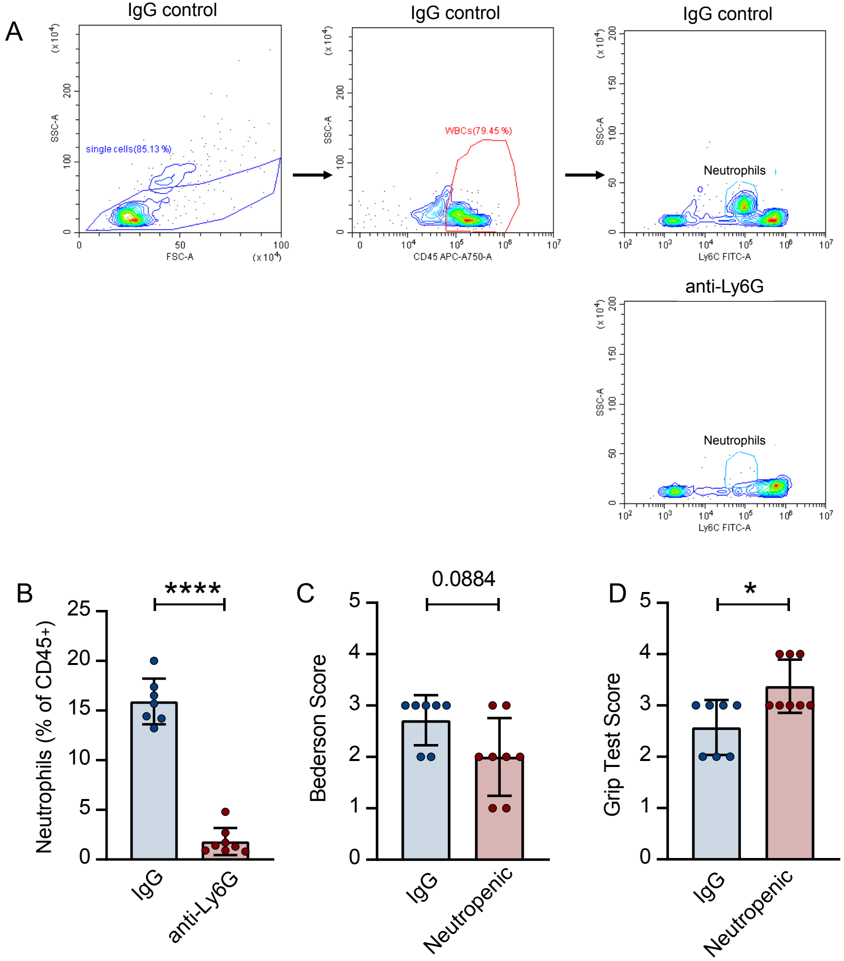

Supplemental Figure 10. Neutrophil depletion improves ischemic stroke outcomes. Mice were subjected to 1 hour of transient middle cerebral artery occlusion followed by 23 hours of reperfusion. One day before stroke onset, mice were injected with a neutrophil-depleting antibody (anti-Ly6G) or IgG control. (A-B) Neutropenia was confirmed by flow cytometry immediately before stroke induction. (C) The Bederson test was used to assess neurological outcome 24 hours after stroke. (D) In parallel, motor function was measured using the grip test. Data was compared between groups using an unpaired t-test (B) or Mann Whitney test (C and D). N=7-8 per group. *P < 0.05, **** P < 0.0001.

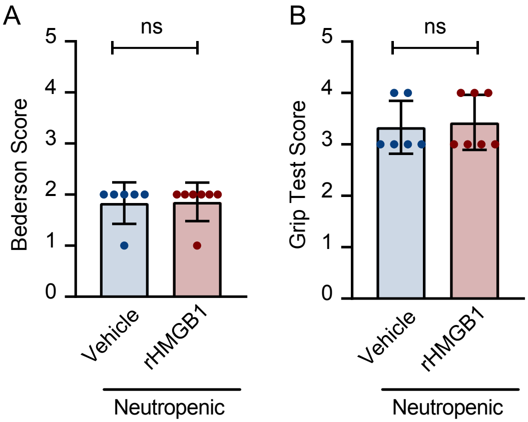

Supplemental Figure 11. Recombinant HMGB1 does not impact stroke outcomes in neutropenic mice. Mice were subjected to 1 hour of transient middle cerebral artery occlusion followed by 23 hours of reperfusion. One day before stroke onset, mice were injected with a neutrophil-depleting antibody (anti-Ly6G) and one hour after stroke onset mice were treated with either recombinant HMGB1 (rHMGB1) or vehicle. (A) The Bederson test was used to assess neurological outcome 24 hours after stroke. (B) In parallel, motor function was measured using the grip test. Data was compared between groups using a Mann Whitney test. N=6-7 per group. Ns = not significantly different.

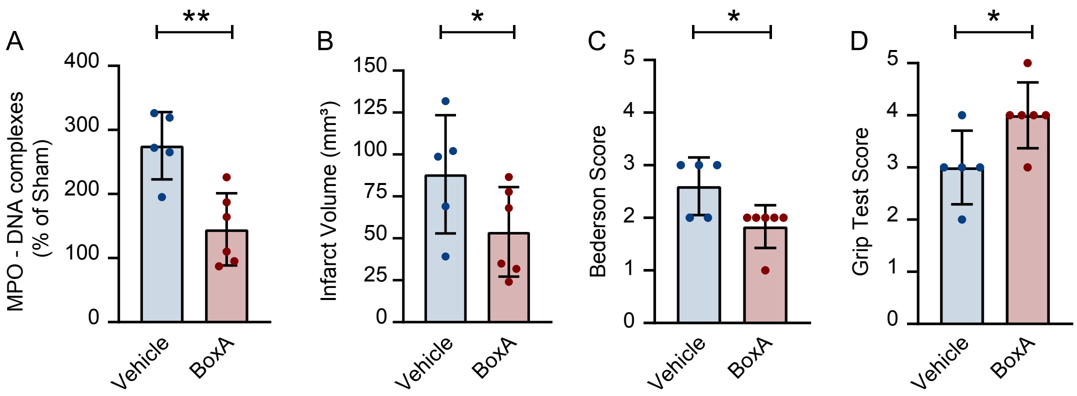

Supplemental Figure 12. Inhibition of HMGB1 with the competitive inhibitor BoxA improves outcomes after stroke. Mice were subjected to 1 hour of transient middle cerebral artery occlusion followed by 23 hours of reperfusion. Prior to ischemic stroke, mice were treated with the HMGB1 inhibitor BoxA (15 mg/kg) or vehicle. Twenty-four hours later, plasma MPO-DNA complexes (A) were assessed as well as brain infarct volume (B). (C) The Bederson test was used to assess neurological outcome 24 hours after stroke. (D) In parallel, motor function was measured using the grip test. Groups were compared using an unpaired T-test (A and B) or Mann-Whitney test (C and D). N=5-6 per group. *P < 0.05; **P < 0.01.

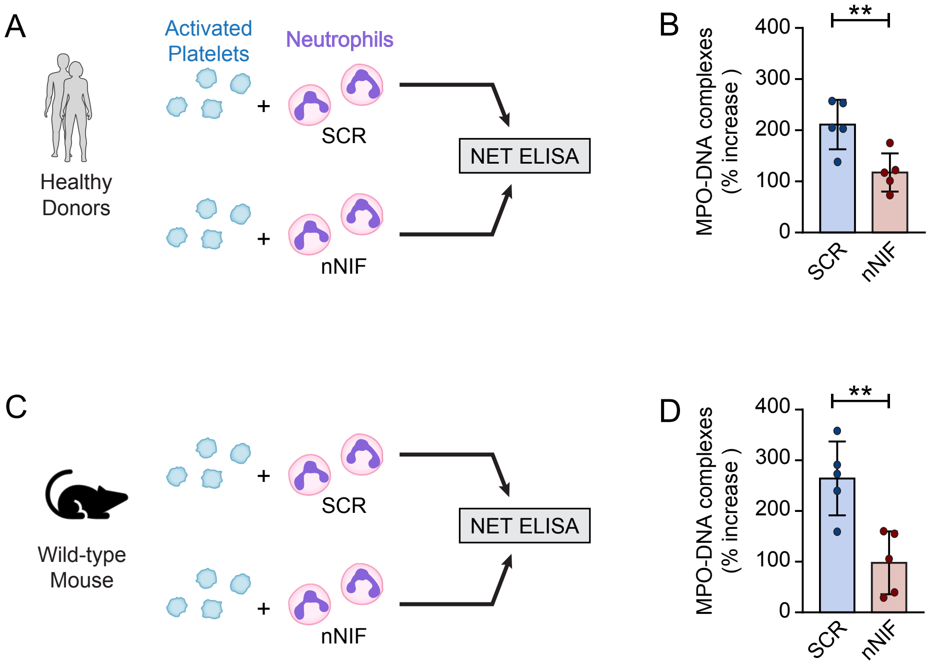

Supplemental Figure 13. nNIF blocks platelet-induced NET formation in human and mouse neutrophils. (A-B) Platelets and neutrophils were isolated from 5 healthy donors. Neutrophils were pre-treated for 1 hour with either nNIF (100nM) or SCR control peptide (100nM). Platelets were activated for 15 minutes with convulxin and then incubated with neutrophils for 180 minutes at 37°C after which NETs were quantified using a MPO-DNA ELISA. (C-D) Platelets and neutrophils were isolated from 5 wildtype mice. Neutrophils were pre-treated for 1 hour with either nNIF (100nM) or SCR control peptide (100nM). Platelets were activated for 15 minutes with convulxin and then incubated with neutrophils for 180 minutes at 37°C after which NETs were quantified using a MPO-DNA ELISA. Groups were compared using an unpaired T-test. N=5 per group. **$P < 0.01$.

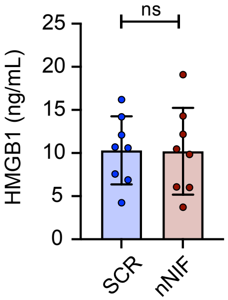

Supplemental Figure 14. nNIF treatment does not reduce HMGB1 release after stroke. Mice were subjected to 1 hour of transient middle cerebral artery occlusion followed by 23 hours of reperfusion. Mice were treated with nNIF or SCR 1 hour before and 1 hour after stroke onset (10 mg/kg). HMGB1 levels were determined in plasma samples obtained 24 hours after stroke onset. Groups were compared using an unpaired T-test. N=8 per group. Ns = not significantly different.

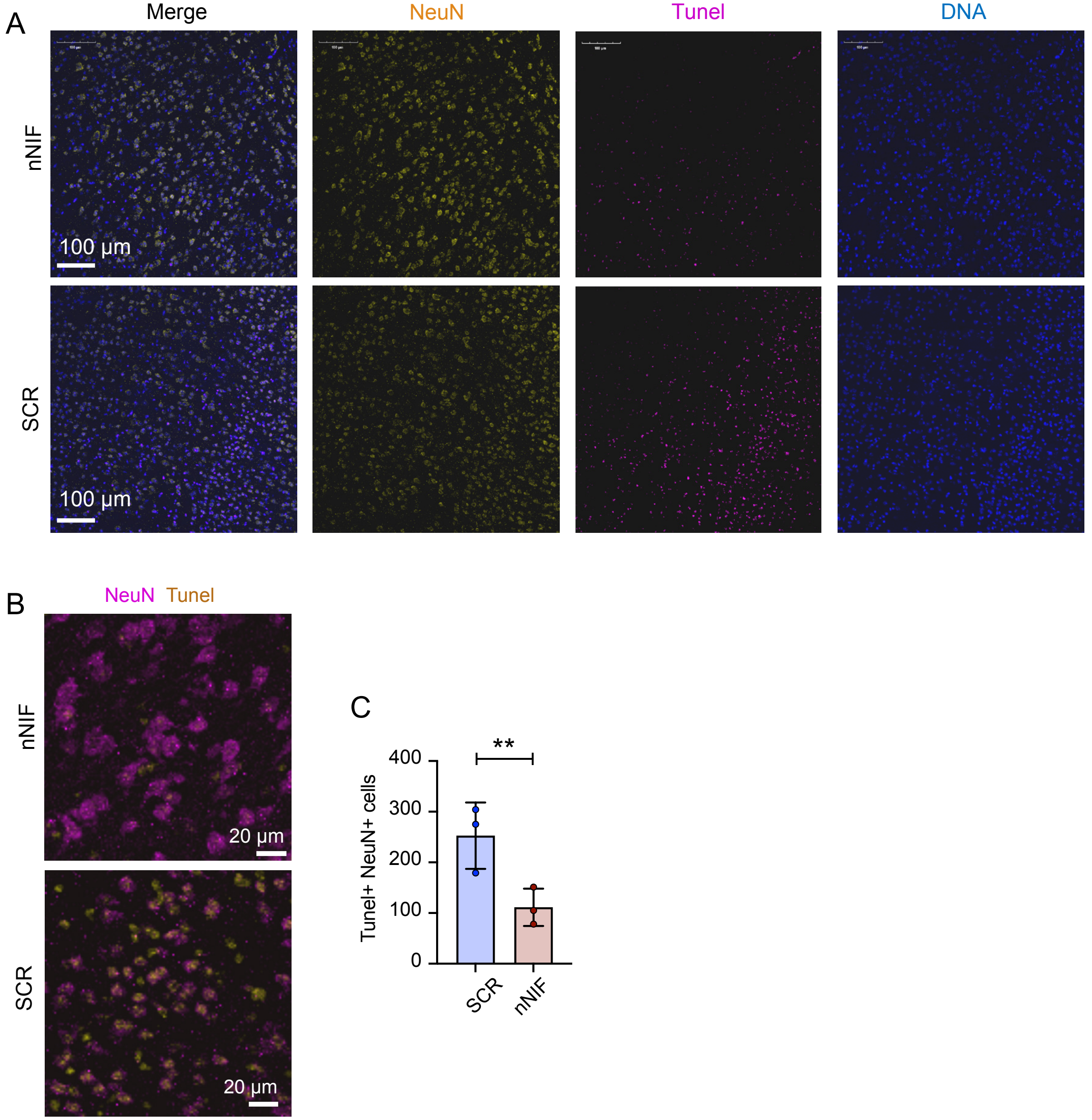

Supplemental Figure 15. nNIF treatment reduces neuronal apoptosis after ischemic stroke. Mice were subjected to 1 hour transient middle cerebral artery occlusion followed by 23 hours of reperfusion. Mice were treated with nNIF or SCR 1 hour before and 1 hour after stroke onset (10 mg/kg). Twenty-four hours after stroke onset, mice were euthanized and brain tissue was dissected and embedded for histology. (A) Brain sections were stained for neuronal apoptosis by labeling DNA-strand breaks with Tunel (magenta) and neurons with NeuN (yellow). DAPI was used as a DNA counterstain (blue). (B) Higher magnification images demonstrating colocalization of Tunel (magenta) and NeuN (yellow). Images are representative for 3 mice in each group. (C) NeuN and Tunel double positive cells were counted in the striatum of nNIF or SCR treated animals. Groups were compared using an unpaired T-test. N = 3. *P < 0.05.

SUPPLEMENTAL VIDEO

Supplemental Video 1. 3D video rendering of Neutrophil Extracellular Traps (NETs) in brain tissue from ischemic stroke patients. NETs were identified by co-localization of myeloperoxidase (MPO; red), citrullinated histone H3 (H3cit; green) and DNA (DAPI; blue). Video is representative of three ischemic stroke patients.

SUPPLEMENTAL TABLES

Supplemental Table 1. Clinical characteristics of the stroke patient cohort.

Characteristics	Healthy Donors (n = 27)	Ischemic Stroke (n = 27)	p-value
Age (mean ± SD; years)	55 ± 15	63 ± 15	0.06
Female (%)	39%	44%	0.99
Diabetes (%)	0%	14.8%	0.11
Hyperlipidemia (%)	0%	40.7%	0.0007
Hypertension (%)	0%	37%	0.0003
Stroke Severity (median (range); NIHSS at admission)	-	3 (0-17)	na
Stroke Outcome (median (range); mRS at discharge)	-	2 (0-5)	na
TOAST subtype			
LAA	-	22%	na
CE	-	15%	na
SVD	-	33%	na
Other	-	11%	na
Unknown	-	19%	na
Medication			
anti-coagulation	-	11%	na
anti-platelets	-	37%	na
thrombolysis	-	22%	na

Supplemental Table 2. Correlations between immunothrombosis markers, stroke severity and stroke outcome. N=27.

	Stroke Severity	Stroke Outcome
**D-Dimers**	r = 0.191; p = 0.305	r = -0.101; p = 0.680
**PF4**	r = -0.052; p = 0.936	r = 0.185; p = 0.410
**Calprotectin**	r = 0.002; p = 0.621	r = -0.109; p = 0.993
**H3cit**	r = 0.225; p = 0.270	r = 0.450; p = 0.024
**MPO-DNA**	r = -0.001; p = 0.995	r = 0.507; p = 0.01
**DNase activity**	r = -0.173; p = 0.418	r = -0.073; p = 0.740
